# Investigations on the Anticancer Potential of Benzothiazole-Based Metallacycles

**DOI:** 10.3389/fchem.2020.00209

**Published:** 2020-04-03

**Authors:** Stephan Mokesch, Klaudia Cseh, Heiko Geisler, Michaela Hejl, Matthias H. M. Klose, Alexander Roller, Samuel M. Meier-Menches, Michael A. Jakupec, Wolfgang Kandioller, Bernhard K. Keppler

**Affiliations:** ^1^Institute of Inorganic Chemistry, Faculty of Chemistry, University of Vienna, Vienna, Austria; ^2^Research Cluster Translational Cancer Therapy Research, University of Vienna, Vienna, Austria; ^3^Department of Analytical Chemistry, Faculty of Chemistry, University of Vienna, Vienna, Austria

**Keywords:** metallacycles, anticancer, benzothiazoles, ruthenium complexes, osmium complexes

## Abstract

A series of 2-phenylbenzothiazole derivatives and their corresponding organometallic ruthenium(II) and osmium(II) complexes were synthesized, designed to exploit both, the attributes of the half-sandwich transition metal scaffold and the bioactivity spectrum of the applied 2-phenylbenzothiazoles. All synthesized compounds were characterized via standard analytical methods. The obtained organometallics showed antiproliferative activity in the low μM range and are thus at least an order of magnitude more potent than the free ligands. ESI-MS measurements showed that the examined compounds were stable in aqueous solution over 48 h. Additionally, their binding preferences to small biomolecules, their cellular accumulation and capacity of inducing apoptosis/necrosis were investigated. Based on the fluorescence properties of the selected ligand and the corresponding ruthenium complex, their subcellular distribution was studied by fluorescence microscopy, revealing a high degree of colocalization with acidic organelles of cancer cells.

## Introduction

**Chart 1:**
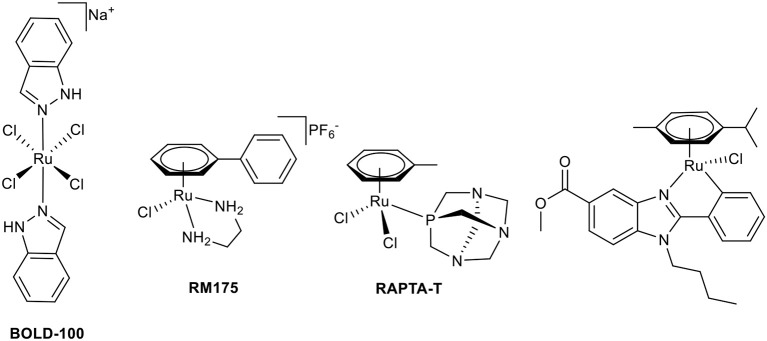
Chemical structures of BOLD-100, RM175, RAPTA-T, and a biologically active *C,N*-chelate half-sandwich Ru(II) complex (Yellol et al., 2015).

The first stepping stone toward the application of coordination compounds for tumor therapy was the discovery of the antiproliferative activity of cisplatin (Rosenberg, 1971, 1973). Soon, other compounds were investigated with the aim of overcoming severe limitations of cisplatin, which include a broad range of side effects, limited spectrum of responsive cancer types, and the development of tumor cell resistances. The first approach was the modification of this original Pt(II) compound (Galanski and Keppler, 2007). However, the scientific community branched out quickly to neighboring transition metals (Jakupec et al., 2008). Promising results have been reported for the first-in-class Ru(III) complex BOLD-100 (formerly IT-139 and KP1339, see Chart 1) in a clinical phase I study (Trondl et al., 2014). The second intensively studied Ru(III) complex is NAMI-A; however, further clinical investigations have been canceled due to insufficient efficacy (Alessio, 2017). Another class of antitumor ruthenium coordination compounds is represented by half-sandwich complexes with piano-stool geometry. Most of these complexes also act as prodrugs, where activation is achieved by hydrolysis of a labile ligand (leaving group). The formed positively charged aqua species are very attractive binding partners for biological nucleophiles, i.e., proteins or DNA. The two most prominent representatives of this compound class are Dyson's RAPTA complexes and Sadler's RAEN organometallics (Chart 1; Bergamo et al., 2012). Our group established organometallic complexes with high antitumor efficacy by utilizing *O,O-*, or *S,O-*chelating bioactive ligands, such as maltol, and *S,O*-chelating ligands, such as thiomaltol, and attempted structure-activity relationship assertions (Kandioller et al., 2009). The drawback of these organometallics is their lowered stability in physiological media which can be increased by exchange of the halido leaving groups, e.g., *N*-heterocyclic nitrogen donors (Hackl et al., 2016; Mokesch et al., 2019).

Another approach to circumvent stability issues is the synthesis of complexes with ligands chelating the transition metal via a heterocyclic nitrogen and an aromatic-carbon atom. This *C,N*-chelating motif has been applied to different transition metals such as Ni(II), Pt(II), Pd(II) (Chen et al., 2008; Cross et al., 2011), Ru(II) (Djukic et al., 2006; Li et al., 2015; Kondrashov et al., 2016), Rh(III), and Ir(III) (Ruiz et al., 2012). The resulting Ru complexes are mostly used as catalysts; e.g., for the stereoselective reduction of ketones (Facchetti et al., 2016), reduction of nitrile functionalities (Nirmala et al., 2016), and hydrogen transfer reactions (Ferrer Flegeau et al., 2011; Yu et al., 2015). In other cases, they are the transition states of the utilized Ru(II) catalyst (Zhang et al., 2014; Rajkumar et al., 2015; Sabater et al., 2016). More recently, Ru complexes of *C,N*-chelating ligands were investigated for anticancer activity (Ruiz et al., 2011; Yellol et al., 2015; Riedl et al., 2017). There is a definite trend toward the exploitation of possible synergistic effects of utilized ligand systems with inherent bioactivity and the potential affinity of the piano-stool configured transition metal complexes toward biological nucleophiles. Pérez et al. synthesized Ru complexes of benzodiazepine ligands (Pérez et al., 2002), Ruiz et al. use *C,N-*chelating conjugates of steroids in Ru(II) and the aforementioned Rh(III)/Ir(III) complexes (Ruiz et al., 2011, 2012). Recently published studies report promising bioactive piano-stool Ru(II) complexes of the pharmacologically widely used benzimidazole scaffold (Chart 1; Belsa et al., 2013; Yellol et al., 2015).

The benzothiazole scaffold has been extensively investigated for non-invasive diagnosis of Alzheimer's disease (Jia et al., 2015) as well as anti-inflammatory (Ban et al., 1998; Papadopoulou et al., 2005), antimicrobial (Singh et al., 2016), anti-allergic (Ban et al., 1998), analgesic (Baell et al., 2001; Westaway et al., 2008), antineoplastic (Chua et al., 2000; Starcevic et al., 2006; Caleta et al., 2009), topoisomerase I/II (Abdel-Aziz et al., 2004; Pinar et al., 2004; Mohmed et al., 2015), and tyrosine kinase inhibiting (Okaniwa et al., 2013; Gabr et al., 2015) properties. Apart from biological applications, *C,N*-coordination of 2-phenylbenzothiazoles to Ir(III) are promising new candidates for the preparation of organic light-emitting diodes (OLEDs), due to their high quantum yield phosphorescence in yellow and orange (Li et al., 2014).

The goal of this study was the synthesis of 2-phenylbenzothiazole-based Ru(II) and Os(II) piano-stool complexes, investigations on their biological activity profiles, and an attempt to derive preliminary structure-activity relationships.

## Materials and Methods

All solvents were purchased from commercial sources and distilled prior use. 2-Aminothiophenol (98%, Aldrich), benzaldehyde (≥98%, Acros), 4-fluorobenzaldehyde (98%, Aldrich), 4-chlorobenzaldehyde (≥98.5%, Acros), 4-methylbenzaldehyde (97%, Acros), 4-methoxybenzaldehyde (≥98%, Fluka), 2-(4-aminophenyl)benzothiazole (98%, Alfa Aesar), ruthenium(III) chloride hydrate (Johnson Matthey), osmium(VIII) tetroxide (Johnson Matthey), hydrazine hydrochloride (>98%, Aldrich), α-terpinene (90%; Acros Organics), and sodium acetate (≥98.5%, Fluka) were purchased and used without further purification. Bis [(η^6^-*p*-isopropyltoluol) dichloridoruthenium(II)] (Bennett and Smith, 1974) and bis [(η^6^-*p*-isopropyltoluol)dichloridoosmium(II)] (Kiel et al., 1990) were synthesized as described by other authors.

Melting points were determined with a *Büchi Melting Point M-560*. The solubility was determined by dissolving the compound in DMF and subsequent dilution to a final concentration of 1% DMF/MEM (minimal essential medium). The highest concentrated dilution, where no precipitation of the compound occurred, was the determined solubility. The NMR spectra were recorded at 25°C using a *Bruker FT-NMR spectrometer Avance III*™ *500 MHz*. ^1^H NMR spectra were measured at 500.10 MHz and ^13^C-NMR Spectra at 125.75 MHz in deuterated chloroform (CDCl_3_). 2D-NMR spectra were measured using a gradient-enhanced mode. CHNS elemental analyses were performed with a *Eurovector EA3000 Elemental Analyzer* in the microanalytical laboratory of the University of Vienna. Single crystals of **L5**, **1a**, and **1b** suitable for X-ray diffraction analysis were grown from DCM/Et_2_O at 4°C. The X-ray intensity data was measured on a Bruker D8 Venture or X8 APEX II diffractometer equipped with multilayer monochromators, Cu K/a INCOATEC micro focus sealed tube and Kryoflex cooling device. The structure was solved by *direct methods* and refined by *full-matrix least-squares techniques*. Non-hydrogen atoms were refined with *anisotropic displacement parameters*. Hydrogen atoms were inserted at calculated positions and refined with a riding model, respectively, as rotating groups. The following software was used: *Bruker SAINT software package* using a narrow-frame algorithm for frame integration, *SADABS* (Sheldrick, 1996) for absorption correction, *OLEX2* (Dolomanov et al., 2009) for structure solution, refinement, molecular diagrams, and graphical user-interface, *Shelxle* (Hubschle et al., 2011) for refinement and graphical user-interface *SHELXS-2013* (Sheldrick, 2015b) for structure solution, *SHELXL-2013* (Sheldrick, 2015a) for refinement*, Platon* (Spek, 2009) for symmetry check. Experimental data and CCDC-code can be found in Table S1. Electronspray ionization mass spectra were recorded on a *Bruker AmaZon SL ion trap mass spectrometer* (Bruker Daltonics GmbH).

### General Procedure for the Synthesis of 4′-Substituted 2-Phenylbenzothiazoles

The appropriate aldehyde (1 eq) was dissolved in ethanol and 2-aminothiophenol (1 eq) was added, followed by slow addition of hydrogen peroxide (30%, 1.8 eq) and HCl (37%, 1.1 eq). After 2.5 h of stirring at room temperature, NaOH (10%) was added until an alkaline solution was obtained. After 10 min in an ice bath, the precipitated product was collected by filtration in good yields (Guo et al., 2009).

### General Complexation Procedure

The appropriate dimeric metal precursor (0.9 eq) and sodium acetate (1.1 eq) were dissolved in MeOH or MeOH:DCM (3:1) and stirred for 1 h at 40°C. After the addition of the desired benzothiazole (1 eq), the reaction mixture is either stirred at 40°C or irradiated under microwave conditions. The solvent was evaporated, the residue taken up in DCM, filtrated, concentrated and the product was then precipitated by addition of *n*-hexane.

#### 2-Phenylbenzothiazole (L1)

The synthesis was performed according to the general procedure for the synthesis of 4′-substituted 2-phenylbenzothiazoles, using benzaldehyde (479 μL, 4.7 mmol), 2-aminothiophenol (504 μL, 4.7 mmol), H_2_O_2_ (866 μL, 8.5 mmol), and HCl (432 μL, 5.2 mmol), yielding an off-white solid (706 mg, 71%). Mp: 111°C, Solubility: 0.02 mg/mL = 0.09 mM (MEM, 1% DMF); ^1^H-NMR (500.10 MHz, d_6_-DMSO) δ: 7.47 [dd, ^3^J(H,H) = 8 Hz, ^3^J(H,H) = 8 Hz, 1H, H5], 7.56 [dd, ^3^J(H,H) = 8 Hz, ^3^J(H,H) = 8 Hz, 1H, H6], 7.58–7.60 (m, 3H, H3′, H4′), 8.07 [d, ^3^J(H,H) = 8 Hz, H7], 8.09–8.12 (m, 2H, H2′), 8.15 [dd, ^3^J(H,H) = 8 Hz, ^4^J(H,H) = 1 Hz, 1H, H4]; ^13^C-NMR (125.75 MHz, d_6_-DMSO) δ: 122.4 (C7), 122.9 (C4), 125.6 (C6), 126.7 (C5), 127.2 (C2′), 129.5 (C4′), 131.5 (C4′), 132.9 (C7a), 134.5 (C1′), 153.6 (C3a), 167.3 (C2); *m/z* 212.23, m_th_: 212.05; elemental analysis calcd. for C_13_H_9_NS: C 73.90, H 4.29, N 6.63, S 15.18%; found: C 73.55, H 4.20, N 6.66, S 15.4 6%.

#### 2-(4-Fluorophenyl)benzothiazole (L2)

The synthesis was performed according to the general procedure for the synthesis of 4′-substituted 2-phenylbenzothiazoles, using 4-fluorobenzaldehyde (505 μL, 4.7 mmol), 2-aminothiophenol (504 μL, 4.7 mmol), H_2_O_2_ (866 μL, 8.5 mmol), and HCl (432 μL, 5.2 mmol), yielding an off-white solid (802 mg, 75%). Mp: 100°C, Solubility: 0.03 mg/mL = 0.13 mM (MEM, 1% DMF); ^1^H-NMR (500.10 MHz, d_6_-DMSO) δ: 7.41 [ddd, ^3^J(H,H) = 9 Hz, ^3^J(H,H) = 9 Hz, ^3^J(H,F) = 2 Hz, 2H, H3′)], 7.46 [ddd, ^3^J(H,H) = 8 Hz, ^3^J(H,F) = 8 Hz, ^4^J(H,H) = 1 Hz, 1H, H5′)], 7.55 [ddd, ^3^J(H,H) = 8 Hz, ^3^J(H,H) = 8 Hz, ^4^J(H,F) = 1 Hz, 1H, H6′)], 8.06 [d, ^3^J(H,H) = 8 Hz, 1H, H7], 8.13–8.17 (m, 3H, H4, H2′)), ^13^C-NMR (125.75 MHz, d_6_-DMSO) δ: 116.5 [d, ^2^J(C,F) = 22 Hz, C3′)], 122.4 (C7), 122.9 (C4), 125.6 (C6), 126.8 (C5), 129.5 [d, ^4^J(C,F) = 3 Hz, C1′)], 129.6 [d, ^3^J(C,F) = 9 Hz, C2′)], 134.6 (C7a), 153.5 (C3a), 163.9 [d, ^1^J(C,F) = 250 Hz, C4′)], 166.1 C2); *m/z* 231.21, mth: 230.04; elemental analysis calcd. for C_13_H_8_FNS·0.1H_2_O: C 67.57, H 3.58, N 6.06, S 13.88%; found: C 67.68, H 3.56, N 6.03, S 13.92%.

#### 2-(4-Chlorophenyl)benzothiazole (L3)

The synthesis was performed according to the general procedure for the synthesis of 4′-substituted 2-phenylbenzothiazoles, using 4-chlorobenzaldehyde (661 mg, 4.7 mmol), 2-aminothiophenol (504 μL, 4.7 mmol), H_2_O_2_ (866 μL, 8.5 mmol), and HCl (432 μL, 5.2 mmol), yielding a purplish gray solid (955 mg, 83%). Mp: 109 °C, Solubility: 0.03 mg/mL = 0.12 mM (MEM, 1% DMF); ^1^H-NMR (500.10 MHz, d_6_-DMSO) δ: 7.49 [dd, ^3^J(H,H) = 8 Hz, ^3^J(H,H) = 8 Hz, 1H, H5], 7.57 [dd, ^3^J(H,H) = 8 Hz, ^3^J(H,H) = 8 Hz, 1H, H6], 7.62–7.66 (m, 2H, H3‘), 8.08 [d, ^3^J(H,H) = 8 Hz, 1H, H7], 8.10–8.14 (m, 2H, H2‘), 8.17 [d, ^3^J(H,H) = 8 Hz, 1H, H4]; ^13^C-NMR (125.75 MHz, d_6_-DMSO) δ: 122.5 (C7), 123.0 (C4), 125.8 (C6), 126.9 (C5), 128.9 (C3′)), 129.5 (C2′)), 131.7 (C1′)), 134.6 (C4′)), 136.1 (C7a), 153.5 (C3a), 166.0 (C2); *m/z* 246.17, m_th_: 246.01; elemental analysis calcd. for C_13_H_8_ClNS·0.1H_2_O: C 63.08, H 3.34, N 5.66, S 12.95%; found: C 62.99, H 3.23, N 5.72, S 13.25%.

#### 2-(4-Methylphenyl)benzothiazole (L4)

The synthesis was performed according to the general procedure for the synthesis of 4′-substituted 2-phenylbenzothiazoles, using 4-tolualdehyde (554 μL, 4.7 mmol), 2-aminothiophenol (504 μL, 4.7 mmol), H_2_O_2_ (866 μL, 8.5 mmol), and HCl (432 μL, 5.2 mmol), yielding a gray solid (897 mg, 85%). Mp: 81°C, Solubility: 0.04 mg/mL = 0.18 mM (MEM, 1% DMF); ^1^H-NMR (500.10 MHz, d_6_-DMSO) δ: 2.37 (s, 3H, H5′)), 7.36 [d, ^3^J(H,H) = 8 Hz, 1H, H3′)], 7.44 [dd, ^3^J(H,H) = 8 Hz, ^3^J(H,H) = 8 Hz, 1H, H5], 7.53 [dd, ^3^J(H,H) = 8 Hz, ^3^J(H,H) = 8 Hz, 1H, H6], 7.97 [d, ^3^J(H,H) = 8 Hz, 1H, H2′)], 8.04 [d, ^3^J(H,H) = 8 Hz, 1H, H7], 8.11 [d, ^3^J(H,H) = 8 Hz, 1H, H4]; ^13^C-NMR (125.75 MHz, d_6_-DMSO) δ: 21.1 (C5′)), 122.3 (C7), 122.7 (C4), 125.4 (C6), 126.6 (C5), 127.2 (C2′)), 130.0 (C3′)), 130.3 (C4′)), 134.3 (C7a), 141.5 (C1′)), 153.6 (C3a), 167.4 (C2); *m/z* 226.24, m_th_: 226.07; elemental analysis calcd. for C_14_H_11_NS·0.1H_2_O: C 74.04, H 4.97, N 6.17, S 14.12%; found: C 73.83, H 4.88, N 6.23, S 14.39%.

#### 2-(4-Methoxyphenyl)benzothiazole (L5)

The synthesis was performed according to the general procedure for the synthesis of 4′-substituted 2-phenylbenzothiazoles, using *p*-anisaldehyde (572 μL, 4.7 mmol), 2-aminothiophenol (504 μL, 4.7 mmol), H_2_O_2_ (866 μL, 8.5 mmol), and HCl (432 μL, 5.2 mmol), yielding a gray solid (929 mg, 82%). Mp: 115°C, Solubility: 0.06 mg/mL = 0.25 mM (MEM, 1% DMF); ^1^H-NMR (500.10 MHz, d_6_-DMSO) δ: 3.85 (s, 3H, H5′)), 7.12 [d, ^3^J(H,H) = 9 Hz, 2H, H3′)], 7.42 [dd, ^3^J(H,H) = 8 Hz, ^3^J(H,H) = 8 Hz, 1H, H5], 7.52 [dd, ^3^J(H,H) = 8 Hz, ^3^J(H,H) = 8 Hz, 1H, H6], 8.01 [d, ^3^J(H,H) = 8 Hz, 1H, H7], 8.03–8.06 (m, 2H, H2′)), 8.10 [d, ^3^J(H,H) = 8 Hz, 1H, H4]; ^13^C-NMR (125.75 MHz, d_6_-DMSO) δ: 55.5 (C5′)), 114.8 (C3′)), 122.3 (C7), 122.5 (C4), 125.2 (C6), 125.6 (C1′)), 126.6 (C5), 129.0 (C2‘), 134.3 (C7a), 153.7 (C3a), 161.9 (C4′)), 167.2 (C2); *m/z* 242.22, m_th_: 242.06; elemental analysis calcd. for C_14_H_11_NOS: C 69.68, H 4.60, N 5.80, S 13.29%; found: C 69.59, H 4.50, N 5.83, S 13.49%.

#### [(Chlorido)(2-phenylbenzothiazolato-κN, κC2′))(η^6^-*p*-cymene)ruthenium(II)] (1a)

The synthesis was performed according to the general complexation procedure, using bis[dichlorido(η^6^-*p*-cymene)ruthenium(II)] (234 mg, 382 μmol), sodium acetate (78 mg, 935 μmol), MeOH:DCM 3:1, 4 mL), and 2-phenylbenzothiazole **L1** (177 mg, 850 μmol). The reaction mixture was stirred at 40°C for 24 h. The product was obtained as red crystals (291 mg, 79%). Mp: >197°C (decomposition), Solubility: 0.1 mg/mL = 0.21 mM (MEM, 1% DMF); ^1^H-NMR (500.10 MHz, CDCl_3_) δ: 0.76 [d, ^3^J(H,H) = 7 Hz, 3H, Hf], 0.91 [d, ^3^J(H,H) = 7 Hz, 3H, Hf], 2.13 (s, 3H, Hg), 2.25 [sept, ^3^J(H,H) = 7 Hz, 1H, He], 5.13 [d, ^3^J(H,H) = 6 Hz, 1H, Hb], 5.38 [d, ^3^J(H,H) = 6 Hz, 1H, Hc], 8.73 [d, ^3^J(H,H) = 6 Hz, 1H, Hc], 5.91 [d, ^3^J(H,H) = 6 Hz, 1H, Hb], 7.08 [dd, ^3^J(H,H) = 8 Hz, ^3^J(H,H) = 8 Hz, 1H, H5′)], 7.23 [dd, ^3^J(H,H) = 8 Hz, ^3^J(H,H) = 8 Hz, 1H, H4′)], 7.45 [dd, ^3^J(H,H) = 8 Hz, ^3^J(H,H) = 8 Hz, 1H, H6], 7.63 [dd, ^3^J(H,H) = 8 Hz, ^3^J(H,H) = 8 Hz, 1H, H5], 7.68 [dd, ^3^J(H,H) = 8 Hz, 4J(H,H) = 1 Hz, 1H, H6′)], 7.86 [d, ^3^J(H,H) = 8 Hz, 1H, H7], 8.26 [d, ^3^J(H,H) = 8 Hz, 1H, H3′)], 8.38 [d, ^3^J(H,H) = 8 Hz, 1H, H4]; ^13^C-NMR (125.75 MHz, CDCl_3_) δ: 19.1 (Cg), 21.8 (Cf), 22.8 (Cf), 31.6 (Ce), 80.6 (Cb), 82.3 (Cc), 90.1 (Cc), 90.7 (Cb), 99.3 (Cd), 102.6 (Ca), 121.3 (C4), 122.9 (C7), 123.1 (C5′)), 125.5 (C6), 126.0 (C6′)), 127.3 (C5), 130.3 (C4′)), 132.2 (C7a), 139.6 (C1′)), 140.0 (C3′)), 151.2 (C3a), 174.6 (C2′)), 183.4 (C2); *m/z* 504.23, m_th_: 504.01; elemental analysis calcd. for C_23_H_22_ClNRuS: C 57.43, H 4.61, N 2.91, S 6.67%; found: C 57.19, H 4.57, N 2.94, S 6.44%.

#### [(Chlorido)(2-(4′)-fluorophenyl)benzothiazolato-κN, κC2′))(η^6^-*p*-cymene)ruthenium(II)] (2a)

The synthesis was performed according to the general complexation procedure, using bis[dichlorido(η^6^-*p*-cymene)ruthenium(II)] (51 mg, 84 μmol), sodium acetate (17 mg, 200 μmol), MeOH:DCM (3:1, 4 mL), and 2-(4-fluorophenyl)benzothiazole **L2** (44 mg, 182 μmol). The reaction mixture was stirred at 40°C for 24 h. The product was obtained as red crystals (68 mg, 82%). Mp: >199°C (decomposition), Solubility: 0.1 mg/mL = 0.20 mM (MEM, 1% DMF); ^1^H-NMR (500.10 MHz, CDCl_3_) δ: 0.77 [d, ^3^J(H,H) = 7 Hz, 3H, Hf], 0.91 [d, ^3^J(H,H) = 7 Hz, 3H, Hf], 2.14 (s, 3H, Hg), 2.25 [sept, ^3^J(H,H) = 7 Hz, 1H, He], 5.15 [dd, ^3^J(H,H) = 6 Hz, ^4^J(H,H) = 1 Hz, 1H, Hb], 5.41 [d, ^3^J(H,H) = 6 Hz, 1H, Hc], 5.70 [d, ^3^J(H,H) = 6 Hz, 1H, Hc], 5.91 [d, ^3^J(H,H) = 6 Hz, 1H, Hb], 6.77 [ddd, ^3^J(H,H) = 6 Hz, ^4^J(H,F) = 3 Hz, ^4^J(H,H) = 1 Hz, 1H, H50′)], 7.45 [dd, ^3^J(H,H) = 8 Hz, ^3^J(H,H) = 8 Hz, 1H, H6], 7.63 [dd, ^3^J(H,H) = 8 Hz, ^3^J(H,H) = 8 Hz, 1H, H5], 7.67 [dd, ^3^J(H,H) = 8 Hz, ^4^J(H,F) = 5 Hz, 1H, H6′)], 7.85 [dd, ^3^J(H,H) = 8 Hz, ^4^J(H,H) = 1 Hz, 1H, H7], 7.94 [dd, ^3^J(H,H) = 8 Hz, ^4^J(H,F) = 2 Hz, 1H, H3′)], 8.33 [d, ^3^J(H,H) = 8 Hz, 1H, H4]; ^13^C-NMR (125.75 MHz, CDCl_3_) δ: 19.1 (Cg), 21.8 (Cf), 22.8 (Cf), 31.1 (Ce), 80.8 (Cb), 82.8 (Cc), 90.1 (Cc), 90.7 (Cb), 99.5 (Cd), 103.1 (Ca), 110.9 [d, ^3^J(C,F) = 24 Hz, C5′)], 121.1 (C4), 123.0 (C7), 125.5 (C6), 125.9 [d, ^3^J(C,F) = 18 Hz, C3′)], 127.4 (C5), 127.4 [^3^J(C,F) = 9 Hz, C6′)], 132.0 (C7a), 135.9 (C1′)), 151.1 (C3a), 162.8 [d, ^3^J(C,F) = 257 Hz, C4′)], 173.6 (C2), 186.6 [d, ^4^J(C,F) = 5 Hz, C2′)]; *m/z* 464.21, m_th_: 464.04; elemental analysis calcd. for C_23_H_21_ClFNRuS: C 55.36, H 4.24, N 2.81, S 6.43%; found: C 55.14, H 4.26, N 2.83, S 6.28%.

#### [(Chlorido)(2-(4′)-chlorophenyl)benzothiazolato-κN, κC2′))(η^6^-*p*-cymene)ruthenium(II)] (3a)

The synthesis was performed according to the general complexation procedure, using bis[dichlorido(η^6^-*p*-cymene)ruthenium(II)] (100 mg, 160 μmol), sodium acetate (33 mg, 400 μmol), and MeOH (4 mL). Before the addition of the ligand, the mixture was irradiated under microwave conditions for 5 min at 40°C. After addition of 2-(4-chlorophenyl)benzothiazole **L3** (89 mg, 360 μmol), the reaction mixture was again irradiated for 10 min at 80°C. The product was obtained as dark red crystals (91 mg, 54%). Mp: >162°C (decomposition), Solubility: 0.04 mg/mL = 0.08 mM (MEM, 1% DMF); ^1^H-NMR (500.10 MHz, CDCl_3_) δ: 0.76 [d, ^3^J(H,H) = 7 Hz, 3H, Hf], 0.91 [d, ^3^J(H,H) = 7 Hz, 3H, Hf], 2.15 (s, 3H, Hg), 2.24 [sept, ^3^J(H,H) = 7 Hz, 1H, He], 5.16 [dd, ^3^J(H,H) = 6 Hz, ^4^J(H,H) = 1 Hz, 1H, Hb], 5.43 [d, ^3^J(H,H) = 6 Hz, 1H, Hc], 5.72 [dd, ^3^J(H,H) = 6 Hz, ^4^J(H,H) = 1 Hz, 1H, Hc], 5.93 [d, ^3^J(H,H) = 6 Hz, 1H, Hb], 7.06 [dd, ^3^J(H,H) = 8 Hz, ^4^J(H,H) = 2 Hz, 1H, H5′)], 7.46 [dd, ^3^J(H,H) = 8 Hz, ^3^J(H,H) = 8 Hz, 1H, H6], 7.59 [d, ^3^J(H,H) = 8 Hz, 1H, H6′)], 7.64 [dd, ^3^J(H,H) = 8 Hz, ^3^J(H,H) = 8 Hz, 1H, H5], 7.86 [dd, ^3^J(H,H) = 8 Hz, ^4^J(H,H) = 1 Hz, 1H, H7], 8.22 [d, ^4^J(H,H) = 2 Hz, 1H, H3′)], 8.35 [d, ^3^J(H,H) = 8 Hz, 1H, H4]; ^13^C-NMR (125.75 MHz, CDCl_3_) δ: 19.2 (Cg), 21.9 (Cf), 22.8 (Cf), 31.1 (Ce), 80.7 (Cb), 83.0 (Cc), 90.0 (Cc), 90.8 (Cb), 99.5 (Cd), 103.2 (Ca), 122.3 (C4), 123.0 (C7), 123.6 (C5′)), 125.7 (C6), 126.6 (C6′)), 127.5 (C5), 132.1 (C7a), 135.9 (C1′)), 138.1 (C4′)), 139.1 (C3′)), 151.0 (C3a), 173.6 (C2′)), 184.9 (C2); *m/z* 538.16, m_th_: 537.97; elemental analysis calcd. for C_23_H_21_Cl_2_NRuS: C 53.59, H 4.11, N 2.72, S 6.22%; found: C 53.32, H 4.08, N 2.77, S 6.11%.

#### [(Chlorido)(2-(4′)-methylphenyl)benzothiazolato-κN, κC2′))(η^6^-*p*-cymene)ruthenium(II)] (4a)

The synthesis was performed according to the general complexation procedure, using bis[dichlorido(η^6^*-p*-cymene)ruthenium(II)] (202 mg, 320 μmol), sodium acetate (70 mg, 800 μmol), and MeOH (4 mL). Before the addition of the ligand, the mixture was irradiated under microwave conditions for 5 min at 40°C. After addition of 2-(4-methylphenyl)benzothiazole **L4** (164 mg, 720 μmol), the reaction mixture was again irradiated for 10 min at 80°C. The product was obtained as green needles (134 mg, 41%). Mp: >187°C (decomposition), Solubility: 0.05 mg/mL = 0.10 mM (MEM, 1% DMF); ^1^H-NMR (500.10 MHz, CDCl_3_) δ: 0.75 [d, ^3^J(H,H) = 7 Hz, 3H, Hf], 0.90 [d, ^3^J(H,H) = 7 Hz, 3H, Hf], 2.13 (s, 3H, Hg), 2.23 [sept, ^3^J(H,H) = 7 Hz, 1H, He], 2.44 (s, 3H, H7′)), 5.13 [dd, ^3^J(H,H) = 6 Hz, ^4^J(H,H) = 1 Hz, 1H, Hb], 5.40 [dd, ^3^J(H,H) = 6 Hz, ^4^J(H,H) = 1 Hz, 1H, Hc], 5.71 [dd, ^3^J(H,H) = 6 Hz, ^4^J(H,H) = 1 Hz, 1H, Hc], 5.91 [d, ^3^J(H,H) = 6 Hz, 1H, Hb], 6.88 [dd, ^3^J(H,H) = 8 Hz, ^4^J(H,H) = 1 Hz, 1H, H5′)], 7.42 [dd, ^3^J(H,H) = 8 Hz, ^3^J(H,H) = 8 Hz, 1H, H6], 7.57 [d, ^3^J(H,H) = 8 Hz, 1H, H6′)], 7.61 [dd, ^3^J(H,H) = 8 Hz, ^3^J(H,H) = 8 Hz, 1H, H5], 7.83 [dd, ^3^J(H,H) = 8 Hz, ^4^J(H,H) = 1 Hz, 1H, H7], 8.08 (s, 1H, H3′)), 8.35 [d, ^3^J(H,H) = 8 Hz, 1H, H4]; ^13^C-NMR (125.75 MHz, CDCl_3_) δ: 19.1 (Cg), 21.9 (Cf), 22.3 (C7‘), 22.8 (Cf), 31.0 (Ce), 80.3 (Cb), 82.5 (Cc), 89.9 (Cc), 90.7 (Cb), 98.8 (Cd), 102.4 (Ca), 121.1 (C4), 122.9 (C7), 124.4 (C5′)), 125.2 (C6), 125.8 (C6′)), 127.2 (C5), 132.1 (C7a), 137.0 (C1′), 140.4 (C3′), 140.5 (C4′), 151.2 (C3a), 174.5 (C2′), 183.4 (C2); *m/z* 518.20, m_th_: 518.03; elemental analysis calcd. for C_24_H_24_ClNRuS·0.75H_2_O: C 56.68, H 5.05, N 2.75, S 6.31%; found: C 56.65, H 4.89, N 2.76, S 6.19%.

#### [(Chlorido)(2-(4′-methoxyphenyl)benzothiazolato-κN, κC2′)(n^6^-*p*-cymene)ruthenium(II)] (5a)

The synthesis was performed according to the general complexation procedure, using bis[dichlorido(η^6^-*p*-cymene)ruthenium(II)] (117 mg, 187 μmol), sodium acetate (38 mg, 460 μmol), MeOH:DCM (3:1, 4 mL), and 2-(4-methoxyphenyl)benzothiazole **L5** (100 mg, 416 μmol). The reaction mixture stirred at 40°C for 24 h. The product was obtained as red crystals (120 mg, 63%). Mp: >148°C (decomposition), Solubility: 0.08 mg/mL = 0.16 mM (MEM, 1% DMF); ^1^H-NMR (500.10 MHz, CDCl_3_) δ: 0.77 [d, ^3^J(H,H) = 7 Hz, 3H, Hf], 0.92 [d, ^3^J(H,H) = 7 Hz, 3H, Hf], 2.13 (s, 3H, Hg), 2.25 [sept, ^3^J(H,H) = 7 Hz, 1H, He], 3.94 (s, 3H, H7′), 5.12 [d, ^3^J(H,H) = 6 Hz, 1H, Hb], 5.38 [d, ^3^J(H,H) = 6 Hz, 1H, Hc], 5.70 [d, ^3^J(H,H) = 6 Hz, 1H, Hc], 5.88 [d, ^3^J(H,H) = 6 Hz, 1H, Hb], 6.63 [dd, ^3^J(H,H) = 8 Hz, ^4^J(H,H) = 2 Hz, 1H, H5′], 7.40 [dd, ^3^J(H,H) = 8 Hz, ^3^J(H,H) = 8 Hz, 1H, H6], 7.59 [dd, ^3^J(H,H) = 8 Hz, ^3^J(H,H) = 8 Hz, 1H, H5], 7.62 [d, ^3^J(H,H) = 8 Hz, 1H, H6′], 7.77 [d, ^4^J(H,H) = 2 Hz, 1H, H3′], 7.81 [dd, ^3^J(H,H) = 8 Hz, ^4^J(H,H) = 1 Hz, 1H, H7], 8.30 [d, ^3^J(H,H) = 8 Hz, 1H, H4]; ^13^C-NMR (125.75 MHz, CDCl_3_) δ: 19.1 (Cg), 21.8 (Cf), 22.9 (Cf), 31.0 (Ce), 55.4 (C7′), 80.6 (Cb), 82.5 (Cc), 90.0 (Cc), 90.5 (Cb), 98.8 (Cd), 102.5 (Ca), 109.8 (C5′), 120.8 (C4), 122.8 (C7), 124.1 (C3′), 124.9 (C6), 127.1 (C6′), 127.3 (C5), 131.9 (C7a), 132.9 (C1′), 151.2 (C3a), 160.3 (C4′), 173.9 (C2′), 185.7 (C2); *m/z* 534.20, mth: 534.02; elemental analysis calcd. for C_24_H_24_ClNORuS: C 56.41, H 4.73, N 2.74, S 6.27%; found: C 56.27, H 4.63, N 2.78, S 6.14%.

#### [(Chlorido)(2-(4′-aminophenyl)benzothiazolato-κN, κC2′)(η^6^-*p*-cymene)ruthenium(II)] (6a)

The synthesis was performed according to the general complexation procedure, using bis[dichlorido(η^6^-*p*-cymene)ruthenium(II)] (451 mg, 737 μmol), sodium acetate (152 mg, 1,810 μmol), MeOH:DCM (3:1, 4 mL), and 2-(4-aminophenyl)benzothiazole **L6** (370 mg, 1,640 μmol). The reaction mixture stirred at 40°C for 24 h under argon. The product was obtained as a green powder (73 mg, 10%). Mp: >222°C (decomposition), Solubility: 0.08 mg/mL = 0.16 mM (MEM, 1% DMF); ^1^H-NMR (500.10 MHz, CDCl_3_) δ: 0.76 [d, ^3^J(H,H) = 7 Hz, 3H, Hf], 0.91 [d, ^3^J(H,H) = 7 Hz, 3H, Hf], 2.12 (s, 3H, Hg), 2.24 [sept, ^3^J(H,H) = 7 Hz, 1H, He], 5.06 [d, ^3^J(H,H) = 6 Hz, 1H, Hb], 5.37 [d, ^3^J(H,H) = 6 Hz, 1H, Hc], 5.67 [d, ^3^J(H,H) = 6 Hz, 1H, Hc], 5.88 [d, ^3^J(H,H) = 6 Hz, 1H, Hb], 6.37 [dd, ^3^J(H,H) = 8 Hz, ^4^J(H,H) = 2 Hz, 1H, H5′], 7.35 [dd, ^3^J(H,H) = 8 Hz, ^3^J(H,H) = 8 Hz, 1H, H6], 7.48 [d, ^3^J(H,H) = 8 Hz, 1H, H6′], 7.52–7.59 (m, 2H, H3′, H5), 7.77 [d, ^3^J(H,H) = 8 Hz, 1H, H7], 8.25 [d, ^3^J(H,H) = 8 Hz, 1H, H4]; ^13^C-NMR (125.75 MHz, CDCl_3_) δ: 19.1 (Cg), 21.9 (Cf), 22.8 (Cf), 31.0 (Ce), 80.2 (Cb), 82.6 (Cc), 89.7 (Cc), 90.5 (Cb), 98.4 (Cd), 102.4 (Ca), 110.8 (C5′), 120.4 (C4), 122.7 (C7), 124.5 (C6), 124.8 (C3′), 127.0 (C5), 127.5 (C6′), 130.6 (C1′), 131.7 (C7a), 148.2 (C4′), 151.3 (C3a), 173.9 (C2′), 185.6 (C2); *m/z* 461.24, m_th_: 461.06; elemental analysis calcd. for C_23_H_23_ClN_2_RuS·0.25H_2_O: C 55.19, H 4.73, N 5.60, S 6.41%; found: C 55.11, H 4.74, N 5.47, S 6.42%.

#### [(Chlorido)(2-phenylbenzothiazolato-κN, κC2′)(η^6^-*p*-cymene)osmium(II)] (1b)

The synthesis was performed according to the general complexation procedure, using bis[dichlorido(η^6^-*p*-cymene)osmium(II)] (100 mg, 130 μmol), sodium acetate (26 mg, 310 μmol), MeOH:DCM 3:1, 4 mL), and 2-phenylbenzothiazole **L1** (60 mg, 280 μmol). The reaction mixture was irradiated with microwaves for 1.5 h at 70°C. The product was obtained after a second precipitation as red crystals (91 mg, 63%). Mp: >228°C (decomposition), Solubility: 0.09 mg/mL = 0.16 mM (MEM, 1% DMF); ^1^H-NMR (500.10 MHz, CDCl_3_) δ: 0.71 [d, ^3^J(H,H) = 7 Hz, 3H, Hf], 0.87 [d, ^3^J(H,H) = 7 Hz, 3H, Hf], 2.16 [sept, ^3^J(H,H) = 7 Hz, 1H, He], 2.27 (s, 3H, Hg), 5.37 [d, ^3^J(H,H) = 5 Hz, 1H, Hb], 5.66 [d, ^3^J(H,H) = 5 Hz, 1H, Hc], 5.75 [d, ^3^J(H,H) = 5 Hz, 1H, Hc], 5.89 [d, ^3^J(H,H) = 5 Hz, 1H, Hb], 7.06 [ddd, d, ^3^J(H,H) = 7 Hz, ^3^J(H,H) = 7 Hz, ^4^J(H,H) = 1 Hz, 1H, H5′], 7.15 [ddd, ^3^J(H,H) = 7 Hz, ^3^J(H,H) = 7 Hz, ^4^J(H,H) = 1 Hz, 1H, H4′], 7.45 [ddd, ^3^J(H,H) = 8 Hz, ^3^J(H,H) = 8 Hz, ^4^J(H,H) = 1 Hz, 1H, H6], 7.60 [ddd, ^3^J(H,H) = 8 Hz, ^3^J(H,H) = 8 Hz, ^4^J(H,H) = 1 Hz, 1H, H5], 7.75 [dd, ^3^J(H,H) = 8 Hz, ^4^J(H,H) = 1 Hz, 1H, H6′], 7.86 [dd, ^3^J(H,H) = 8 Hz, ^4^J(H,H) = 1 Hz, 1H, H7], 8.12 [d, ^3^J(H,H) = 8 Hz, 1H, H3′], 8.17 [d, ^3^J(H,H) = 8 Hz, 1H, H4]; ^13^C-NMR (125.75 MHz, CDCl_3_) δ: 19.0 (Cg), 22.1 (Cf), 23.2 (Cf), 31.4 (Ce), 69.7 (Cb), 73.2 (Cc), 80.7 (Cc), 80.8 (Cb), 89.6 (Cd), 96.8 (Ca), 121.7 (C4), 123.0 (C5′), 123.0 (C7), 125.6 (C6), 125.8 (C6′), 127.4 (C5), 131.3 (C3′), 132.0 (C7a), 139.7 (C4′), 139.9 (C1′), 150.9 (C3a), 169.0 (C2′), 178.1 (C2); *m/z* 594.26, m_th_: 594.07; elemental analysis calcd. for C_23_H_22_ClNOsS: C 48.45, H 3.89, N 2.46, S 5.62%; found: C 48.25, H 3.86, N 2.52, S 5.55%.

#### [(Chlorido)(2-(4′-fluorophenyl)benzothiazolato-κN, κC2′)(η^6^-*p*-cymene)osmium(II)] (2b)

The synthesis was performed according to the general complexation procedure, using bis[dichlorido(η^6^-*p*-cymene)osmium(II)] (200 mg, 225 μmol), sodium acetate (51 mg, 620 μmol), and MeOH (4 mL). Before the addition of the ligand, the mixture was irradiated under microwave conditions for 5 min at 40°C. After addition of 2-(4-fluorophenyl)benzothiazole **L2** (130 mg, 562 μmol), the reaction mixture was again irradiated for 20 min at 90°C. The product was obtained as a gray powder (124 mg, 42%). Mp: >227°C (decomposition), Solubility: 0.10 mg/mL = 0.17 mM (MEM, 1% DMF); ^1^H-NMR (500.10 MHz, CDCl_3_) δ: 0.72 [d, ^3^J(H,H) = 7 Hz, 3H, Hf], 0.88 [d, ^3^J(H,H) = 7 Hz, 3H, Hf], 2.17 [sept, ^3^J(H,H) = 7 Hz, 1H, He], 2.27 (s, 3H, Hg), 5.37 [d, ^3^J(H,H) = 5 Hz, 1H, Hb], 5.68 [d, ^3^J(H,H) = 5 Hz, 1H, Hc], 5.72 [d, ^3^J(H,H) = 5 Hz, 1H, Hc], 5.88 [d, ^3^J(H,H) = 5 Hz, 1H, Hb], 6.77 [ddd, d, ^3^J(H,H) = 8 Hz, ^4^J(H,H) = 2 Hz, ^4^J(H,F) = 2 Hz, 1H, H5′], 7.45 [dd, ^3^J(H,H) = 8 Hz, ^3^J(H,H) = 8 Hz, 1H, H6], 7.61 [dd, ^3^J(H,H) = 8 Hz, ^3^J(H,H) = 8 Hz, 1H, H5], 7.71–7.80 (m, 2H, H3', H6'), 7.85 [d, ^3^J(H,H) = 8 Hz, 1H, H7], 8.13 [d, ^3^J(H,H) = 8 Hz, 1H, H4]; ^13^C-NMR (125.75 MHz, CDCl_3_) δ: 19.0 (Cg), 22.1 (Cf), 23.2 (Cf), 31.4 (Ce), 70.0 (Cb), 73.8 (Cc), 80.7 (Cc), 80.8 (Cb), 90.1 (Cd), 97.3 (Ca), 110.8 [d, ^3^J(C,F) = 24 Hz, 1C, C5′], 121.6 (C4), 123.0 (C7), 125.4 [d, ^3^J(C,F) = 17 Hz, 1C, C3′], 125.6 (C6), 127.5 [d, ^3^J(C,F) = 9 Hz, 1C, C6′], 127.5 (C5), 131.8 (C7a), 136.4 [d, ^4^J(C,F) = 2 Hz, 1C, C1′], 150.9 (C3a), 164.0 [d, ^1^J(C,F) = 256 Hz, 1C, C4'], 171.8 [d, ^3^J(C,F) = 6 Hz, 1C, C2'], 176.9 (C2); *m/z* 612.25, m_th_: 612.06; elemental analysis calcd. for C_23_H_21_ClFNOsS·0.1H_2_O: C 46.82, H 3.62, N 2.37, S 5.44%; found: C 46.49, H 3.70, N 2.31, S 5.15%.

#### [(Chlorido)(2-(4′-chlorophenyl)benzothiazolato-κN, κC2′)(η^6^-*p*-cymene)osmium(II)] (3b)

The synthesis was performed according to the general complexation procedure, using bis[dichlorido(η^6^-*p*-cymene)osmium(II)] (250 mg, 316 μmol), sodium acetate (63 mg, 772 μmol), and MeOH (4 mL). Before the addition of the ligand, the mixture was irradiated under microwave conditions for 5 min at 40°C. After addition of 2-(4-chlorophenyl)benzothiazole **L3** (172 mg, 702 μmol), the reaction mixture was again irradiated for 20 min at 90°C. The product was obtained as orange crystals (87 mg, 23%). Mp: >202°C (decomposition), Solubility: 0.04 mg/mL = 0.07 mM (MEM, 1% DMF); ^1^H-NMR (500.10 MHz, CDCl_3_) δ: 0.71 [d, ^3^J(H,H) = 7 Hz, 3H, Hf], 0.88 [d, ^3^J(H,H) = 7 Hz, 3H, Hf], 2.16 [sept, ^3^J(H,H) = 7 Hz, 1H, He], 2.28 (s, 3H, Hg), 5.39 [d, ^3^J(H,H) = 5 Hz, 1H, Hb], 5.70 [d, ^3^J(H,H) = 5 Hz, 1H, Hc], 5.74 [d, ^3^J(H,H) = 5 Hz, 1H, Hc], 5.89 [d, ^3^J(H,H) = 5 Hz, 1H, Hb], 7.04 [dd, ^3^J(H,H) = 8 Hz, ^4^J(H,H) = 2 Hz, 1H, H5′], 7.46 [ddd, ^3^J(H,H) = 8 Hz, ^3^J(H,H) = 8 Hz, ^4^J(H,H) = 1 Hz, 1H, H6], 7.61 [ddd, ^3^J(H,H) = 8 Hz, ^3^J(H,H) = 8 Hz, ^4^J(H,H) = 1 Hz, 1H, H5], 7.67 [d, ^3^J(H,H) = 8 Hz, 1H, H6'], 7.86 [dd, ^3^J(H,H) = 8 Hz, ^4^J(H,H) = 1 Hz, 1H, H7], 8.08 [d, ^4^J(H,H) = 2 Hz, 1H, H3'], 8.17 [d, ^3^J(H,H) = 8 Hz, 1H, H4]; ^13^C-NMR (125.75 MHz, CDCl_3_) δ: 19.0 (Cg), 22.1 (Cf), 23.2 (Cf), 31.4 (Ce), 69.8 (Cb), 73.9 (Cc), 80.7 (Cc), 80.8 (Cb), 90.1 (Cd), 97.4 (Ca), 121.7 (C4), 123.1 (C7), 123.4 (C5'), 125.8 (C6), 126.6 (C6′), 127.6 (C5), 131.9 (C7a), 137.1 (C1′), 138.5 (C4′), 139.0 (C3′), 150.9 (C3a), 170.3 (C2′), 177.0 (C2); *m/z* 628.20, m_th_: 628.03; elemental analysis calcd. for C_23_H_21_Cl_2_NOsS: C 45.69, H 3.50, N 2.32, S 5.30%; found: C 45.75, H 3.46, N 2.29, S 5.27%.

#### [(Chlorido)(2-(4′-methylphenyl)benzothiazolato-κN, κC2′)(η^6^-*p*-cymene)osmium(II)] (4b)

The synthesis was performed according to the general complexation procedure, using bis[dichlorido(η^6^-*p*-cymene)osmium(II)] (120 mg, 150 μmol), sodium acetate (30 mg, 370 μmol), and MeOH (4 mL). Before the addition of the ligand, the mixture was irradiated under microwave conditions for 5 min at 40°C. After addition of 2-(4-methylphenyl)benzothiazole **L4** (76 mg, 340 μmol), the reaction mixture was again irradiated for 25 min at 90°C. The product was obtained as yellow needles (109 mg, 62%). Mp: >192°C (decomposition), Solubility: 0.03 mg/mL = 0.05 mM (MEM, 1% DMF); ^1^H-NMR (500.10 MHz, CDCl_3_) δ: 0.71 [d, ^3^J(H,H) = 7 Hz, 3H, Hf], 0.87 [d, ^3^J(H,H) = 7 Hz, 3H, Hf], 2.15 [sept, ^3^J(H,H) = 7 Hz, 1H, He], 2.27 (s, 3H, Hg), 2.43 (s, 3H, H7′), 5.36 [d, ^3^J(H,H) = 5 Hz, 1H, Hb], 5.67 [d, ^3^J(H,H) = 5 Hz, 1H, Hc], 5.73 [d, ^3^J(H,H) = 5 Hz, 1H, Hc], 5.87 [d, ^3^J(H,H) = 5 Hz, 1H, Hb], 6.88 [d, ^3^J(H,H) = 8 Hz, 1H, H5′], 7.42 [dd, ^3^J(H,H) = 8 Hz, ^3^J(H,H) = 8 Hz, 1H, H6], 7.58 [dd, ^3^J(H,H) = 8 Hz, ^3^J(H,H) = 8 Hz, 1H, H5], 7.64 [d, ^3^J(H,H) = 8 Hz, 1H, H6′], 7.83 [d, ^3^J(H,H) = 8 Hz, 1H, H7], 7.93 (s, 1H, H3'), 8.13 [d, ^3^J(H,H) = 8 Hz, 1H, H4]; ^13^C-NMR (125.75 MHz, CDCl_3_) δ: 19.0 (Cg), 22.1 (Cf), 22.2 (C7′),23.2 (Cf), 31.4 (Ce), 69.4 (Cb), 73.4 (Cc), 80.5 (Cc), 80.6 (Cb), 89.3 (Cd), 96.7 (Ca), 121.5 (C4), 122.9 (C7), 124.3 (C5'), 125.3 (C6), 125.6 (C6′), 127.3 (C5), 131.8 (C7a), 137.4 (C1′), 140.1 (C3′), 141.6 (C4′), 150.9 (C3a), 169.1 (C2′), 178.0 (C2); *m/z* 608.26, m_th_: 608.08; elemental analysis calcd. for C_24_H_24_ClNOsS·0.5H_2_O: C 48.59, H 4.25, N 2.36, S 5.41%; found: C 48.38, H 4.11, N 2.26, S 5.10%.

#### [(Chlorido)(2-(4′-methoxyphenyl)benzothiazolato-κN, κC2′)(η^6^-*p*-cymene)osmium(II)] (5b)

The synthesis was performed according to the general complexation procedure, using bis[dichlorido(η^6^-*p*-cymene)osmium(II)] (150 mg, 190 μmol), sodium acetate (38 mg, 465 μmol), and MeOH (4 mL). Before the addition of the ligand, the mixture was irradiated under microwave conditions for 5 min at 40°C. After addition of 2-(4-methoxyphenyl)benzothiazole **L5** (102 mg, 422 μmol), the reaction mixture was again irradiated for 30 min at 90°C. The product was obtained as brown needles (74 mg, 32%). Mp: >137°C (decomposition), Solubility: 0.02 mg/mL = 0.03 mM (MEM, 1% DMF); ^1^H-NMR (500.10 MHz, CDCl_3_) δ: 0.72 [d, ^3^J(H,H) = 7 Hz, 3H, Hf], 0.88 [d, ^3^J(H,H) = 7 Hz, 3H, Hf], 2.16 [sept, ^3^J(H,H) = 7 Hz, 1H, He], 2.27 (s, 3H, Hg), 3.93 (s, 3H, H7'), 5.35 [d, ^3^J(H,H) = 5 Hz, 1H, Hb], 5.66 [d, ^3^J(H,H) = 5 Hz, 1H, Hc], 5.71 [d, ^3^J(H,H) = 5 Hz, 1H, Hc], 5.86 [d, ^3^J(H,H) = 5 Hz, 1H, Hb], 6.63 [dd, ^3^J(H,H) = 8 Hz, ^4^J(H,H) = 2 Hz, 1H, H5'], 7.40 [ddd, ^3^J(H,H) = 8 Hz, ^3^J(H,H) = 8 Hz, ^4^J(H,H) = 1 Hz, 1H, H6], 7.56 [ddd, ^3^J(H,H) = 8 Hz, ^3^J(H,H) = 8 Hz, ^4^J(H,H) = 1 Hz, 1H, H5], 7.61 [d, ^4^J(H,H) = 2 Hz, 1H, H3′], 7.70 [d, ^3^J(H,H) = 8 Hz, 1H, H6'], 7.81 [d, ^3^J(H,H) = 8 Hz, 1H, H7′], 8.09 [d, ^3^J(H,H) = 8 Hz, 1H, H4]; ^13^C-NMR (125.75 MHz, CDCl_3_) δ: 18.9 (Cg), 22.1 (Cf), 23.2 (Cf), 31.4 (Ce), 55.4 (C7′), 69.7 (Cb), 73.5 (Cc), 80.4 (Cc), 80.5 (Cb), 89.4 (Cd), 96.8 (Ca), 109.9 (C4′), 121.2 (C4), 122.9 (C7), 123.5 (C5′), 125.0 (C6), 127.2 (C6′), 127.3 (C5), 131.7 (C7a), 133.4 (C1′), 151.0 (C3a), 161.5 (C4′), 171.2 (C2′), 177.2 (C2); *m/z* 624.24, m_th_: 624.08; elemental analysis calcd. for C_24_H_24_ClNOOsS·H_2_O: C 46.63, H 4.24, N 2.27, S 5.19%; found: C 46.66, H 3.92, N 2.29, S 5.06%.

#### [(Chlorido)(2-(4′-aminophenyl)benzothiazolato-κN, κC2′)(η^6^-*p*-cymene)osmium(II)] (6b)

The synthesis was performed according to the general complexation procedure, using bis[dichlorido(η^6^-*p*-cymene)osmium(II)] (100 mg, 130 μmol), sodium acetate (26 mg, 310 μmol), and MeOH (4 mL). Before the addition of the ligand, the mixture was irradiated under microwave conditions for 5 min at 40°C. After addition of 2-(4-aminophenyl)benzothiazole **L6** (64 mg, 280 μmol), the reaction mixture was again irradiated for 45 min at 90°C. The product was obtained as a green powder (114 mg, 77%). Mp: >134°C (decomposition), Solubility: 0.06 mg/mL = 0.10 mM (MEM, 1% DMF); ^1^H-NMR (500.10 MHz, CDCl_3_) δ: 0.72 [d, ^3^J(H,H) = 7 Hz, 3H, Hf], 0.88 [d, ^3^J(H,H) = 7 Hz, 3H, Hf], 2.14 [sept, ^3^J(H,H) = 7 Hz, 1H, He], 2.26 (s, 3H, Hg), 4.00 (s, 2H, H7'), 5.30 [d, ^3^J(H,H) = 5 Hz, 1H, Hb], 5.64 [d, ^3^J(H,H) = 5 Hz, 1H, Hc], 5.68 [d, ^3^J(H,H) = 5 Hz, 1H, Hc], 5.85 [d, ^3^J(H,H) = 5 Hz, 1H, Hb], 6.38 [dd, ^3^J(H,H) = 8 Hz, ^4^J(H,H) = 2 Hz, 1H, H5'], 7.37 [d, ^4^J(H,H) = 2 Hz, 1H, H3′], 7.54 [dd, ^3^J(H,H) = 8 Hz, ^3^J(H,H) = 8 Hz, 1H, H5], 7.76 [d, ^3^J(H,H) = 8 Hz, 1H, H6′], 7.89 [d, ^3^J(H,H) = 8 Hz, 1H, H7], 8.03 [d, ^3^J(H,H) = 8 Hz, 1H, H4]; ^13^C-NMR (125.75 MHz, CDCl_3_) δ: 18.9 (Cg), 22.1 (Cf), 23.2 (Cf), 31.4 (Ce), 69.3 (Cb), 73.6 (Cc), 80.2 (Cc), 80.4 (Cb), 88.8 (Cd), 96.6 (Ca), 110.8 (C5′), 120.8 (C4), 122.7 (C7), 124.3 (C3′), 124.6 (C6), 127.1 (C5), 127.5 (C6′), 131.1 (C1′), 131.4 (C7a), 149.5 (C4′), 151.0 (C3a), 171.1 (C2′), 177.2 (C2); *m/z* 609.25, m_th_: 609.08; elemental analysis calcd. for C_23_H_23_ClN_2_OsS·0.5H_2_O: C 46.49, H 4.07, N 4.71, S 5.40%; found: C 46.30, H 4.07, N 4.48, S 5.02%.

### ESI-MS Experiments

Electrospray ionization mass spectra were recorded on a *Bruker AmaZon SL ion trap mass spectrometer* (Bruker Daltonics GmbH). The data was acquired and processed using Compass 1.3 and Data Analysis 4.0 software packages (Bruker Daltonics GmbH). Protein spectra were deconvoluted by applying the maximum entropy algorithm with a *m/z* 0.2 mass step and a *m/z* 0.5 instrument peak width. The mass spectra were recorded by direct infusion with a sample concentration of 5 μM and a flow rate of 240 μL/h. Amino acids and nucleoside triphosphates were measured with the following settings: dry temperature 180°C, nebulizer 8.00 psi, dry gas 6 L/min, and the high voltage capillary was set to ±4.5 kV for positive and negative modes and the target mass was set to *m/z* 600. Proteins were measured with the following settings: dry temperature 180°C, nebulizer 6.00 psi, dry gas 6.00 L/min, the high voltage capillary was set to −3.5 kV and the target mass was set to *m/z* 1,000. Amino acids, nucleoside triphosphates and stability measurement samples were diluted with water: methanol mixture (50: 50), whereas protein samples were diluted with water: methanol: formic acid (49.9: 49.9: 0.2). The stock solutions of the ligands and complexes were prepared in DMF and diluted to a final concentration of 1% DMF in H_2_O. The molar ratios used for the interaction studies were ligand/complex: l-histidine: l-cysteine: l-methionine (1: 1: 1: 1), ligand/complex*: N*-Ac-l-cysteine: *N*-Ac-l-histidine: *N*-Ac-l-methionine: and Se-methyl-l-cysteine (1: 1: 1: 1: 1), ligand/complex: Se-l-cystine: DTT (1: 1: 1), ligand/complex: 5'-ATP: 5'-GTP (1: 1: 1), ligand/complex: amino acids: nucleoside triphosphates (1: 1: 1) and ligand/complex: cytochrome-c: ubiquitin (1: 1: 1). The mass spectra were recorded after 1, 3, 6, 24, and 48 h of incubation at 37°C. The relative intensities described in the discussion section refer to the percent peak area of all visible ruthenium and osmium adducts in the spectrum.

### Cell Culture

CH1/PA-1 (ovarian teratocarcinoma) cells were a generous gift from Lloyd R. Kelland (CRC Center for Cancer Therapeutics, Institute of Cancer Research, Sutton, UK). A549 and SW480 cells were kindly provided by the Institute of Cancer Research, Department of Medicine I, Medical University of Vienna, Austria. All cell lines were grown as monolayer cultures in Eagle's minimal essential medium (MEM) supplemented with l-glutamine (4 mM), sodium pyruvate (1 mM), and 1% (v/v) non-essential amino acid solution (all from Sigma-Aldrich) and 10% (v/v) heat-inactivated fetal calf serum (FCS, from BioWest) in 75 cm^2^ flasks (Starlab) at 37°C under a humidified atmosphere containing 5% CO_2_ in air.

### MTT Assay

Antiproliferative activity of the compounds was determined with the colorimetric MTT assay (MTT = 3-(4,5-dimethyl-2-thiazolyl)-2,5-diphenyl-2H-tetrazolium bromide). 1 × 10^3^ CH1/PA-1, 2 × 10^3^ SW480 and 3 × 10^3^ A549 cells were seeded in 100 μL per well into 96-well microculture plates (Starlab). After 24 h, test compounds were dissolved in DMF (Acros), serially diluted in complete MEM not to exceed a final DMF content of 0.5% v/v and added in 100 μL per well. After 96 h, the drug-containing medium was replaced with 100 μL of RPMI 1640/MTT mixture [6 parts of RPMI 1640 medium (Sigma-Aldrich; supplemented with 10% heat-inactivated fetal bovine serum and 4 mM l-glutamine), 1 part of MTT solution in phosphate-buffered saline (5 mg/mL; both from Sigma-Aldrich)]. After incubation for 4 h, the MTT-containing medium was replaced with 150 μL DMSO per well to dissolve the formazan product formed by viable cells. Optical densities at 550 nm (and at the reference wavelength of 690 nm) were measured with a microplate reader (ELx808, Bio-Tek). The 50% inhibitory concentrations (IC_50_) relative to untreated controls were interpolated from concentration-effect curves. At least three independent experiments were performed, each with triplicates per concentration level.

### Flow Cytometry

Compounds were studied for their capacity of inducing programmed cell death (apoptosis) or necrosis by the flow-cytometric annexin V/propidium iodide (PI) assay. SW480 cells were harvested by using trypsin and 7 × 10^4^ cells were placed into 24-well culture plates (Starlab) in 600 μL of complete MEM per well. After 24 h preincubation, cells were treated with different concentrations of the test compounds. The 20 mM stock solutions of the test compounds in DMF were diluted with MEM so that the final DMF concentration never exceeded 0.5% v/v of the treatment solution to avoid DMF toxicity. After incubation for 24 h at 37°C under 5% CO_2_, the media were aspirated and collected; cells were washed once with 37°C warm PBS, trypsinised with TrypLE^TM^ Express (Thermo Fisher) and mixed with the corresponding supernatant. Cell pellets were obtained by centrifugation (300 g, 3 min) and the supernatants were discarded. Then, cells were resuspended with FITC-conjugated annexin V (0.25 μg/mL; BioVision) in binding buffer (10 mM HEPES/NaOH, pH 7.4, 140 mM NaCl, 2.5 mM CaCl_2_), incubated at 37°C for 15 min and stained with PI (1 μg/mL; Fluka) shortly before measurement with a Millipore Guava easyCyte 8HT flow cytometer and InCyte software. The resulting dot plots were analyzed with FlowJo software (TreeStar). All experiments were performed at least three times independently using the same conditions, and each comprised of three replicates.

### Cellular Accumulation

Accumulation of selected compounds in SW480 cells was determined analogous to a method recently developed for the quantification of Os in biological samples (Klose et al., 2017), which was applied here to Ru as well for reasons of comparability. 1.8 × 10^5^ cells per well were seeded in 1 mL of complete MEM into 12-well plates (Starlab) and incubated at 37°C for 24 h. Test compounds were dissolved in DMF, diluted to 50 μM (0.5% DMF) with complete MEM and added in 500 μL per well upon removal of the medium that had been used for seeding. After exposure for 2 h at 37°C, the medium was removed and cells were washed three times with phosphate buffered saline (PBS; Sigma-Aldrich) and lyzed with 400 μL of an adjusted radioimmunoprecipitation assay (RIPA) buffer (150 mM NaCl, 1.0% Triton X-100, 0.1% SDS, and 50 mM Tris, pH 8.0) on a shaker for 2 h at 4°C. Residues were scraped off the wells, and 350 μL of lysates were transferred into 1.5 mL microtubes and centrifuged at 14,000 rpm for 10 min at 4°C. Three hundred microliter of lysates were diluted with a stabilization solution (ascorbic acid, thiourea, EDTA, each 0.5 mM in water, and HNO_3_ as appropriate to yield a final content of 3%) to final volumes of 8 mL. Ru and Os were quantified by ICP-MS with an Agilent 7500ce instrument equipped with a CETAC ASX-520 autosampler and a MicroMist nebulizer at a sample uptake rate of ~0.25 mL/min. The Agilent MassHunter software package (Workstation Software, version B.01.01, Build 123.11, Patch 4, 2012) was used for data processing. Nickel was used as cone material. The experimental parameters were as follows: 1,560 W RF power, 0.92–0.97 L/min carrier gas, 0.22–0.27 L/min make up gas, 15 L/min plasma gas, 0.3 s dwell time and 10 replicates each with 100 sweeps. Results are the means of at least three independent experiments. The ICP-MS was tuned on a daily basis to achieve maximum sensitivity. Additional instrument parameters can be found in Table S11.

### Fluorescence Microscopy

SW480 cells were seeded on coverslips in 6-well plates (1.5 × 10^5^ cells per well in 2 mL of MEM) and allowed to attach overnight. Cells were incubated with **L6** and **6a** (50 μM, 60–120 min) in MEM and stained with LysoTracker Red (Invitrogen) (1 μM, 5 min). After removing the medium and washing with PBS (1×), the cells were treated with fixing solution (2 % paraformaldehyde in PBS) for 5 min and washed with PBS (1×). A BX40 fluorescence microscope with an F-View CCD camera, Cell∧F fluorescence imaging software, and a 60× magnification oil immersion objective lens (all from Olympus) were used for the visualization of compound distribution. The green fluorescence signal of compounds **L6** and **6a** upon excitation at wavelengths of 330–395 nm as well as the red fluorescence signal of LysoTracker Red upon excitation at wavelengths of 510–550 nm were recorded. The exposure time (100 ms) for both channels was kept as short as possible to minimize bleaching. The recorded images were colocalized by means of ImageJ 1.52a and GIMP 2.10.12 software. The correlation analysis of fluorescence images was performed by using Fiji ImageJ software (https://imagej.net/Fiji/Downloads). To evaluate the degree of colocalization of the green (drug) and red (LysoTracker) signals, Pearson's correlation coefficients were calculated (ImageJ, Coloc2 plugin). For this purpose, regions of interest (ROIs) were selected to delineate single cells for analysis (n = 15 for each sample).

## Results and Discussion

**Scheme 1:**
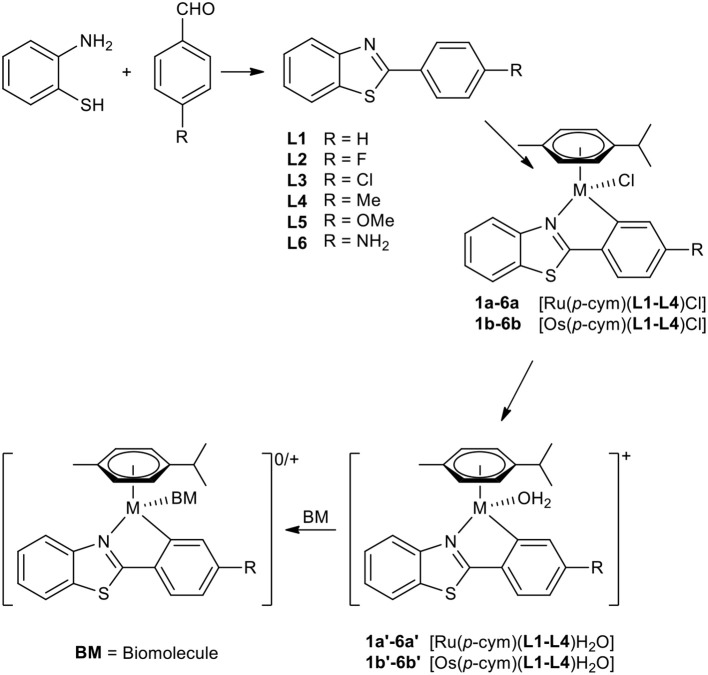
Synthesis of compounds **1a−6b** and the supposed behavior in biological media.

A set of six 4'-substituted 2-phenylbenzothiazoles and their respective Ru(II) and Os(II) complexes was synthesized (Scheme 1). The ligands were prepared by facilitating an acid catalyzed nucleophilic attack of the amino group of 2-aminothiophenol on the carbonyl carbon of the appropriate 4-substituted benzaldehyde, followed by oxidative ring closure to form the desired product in good yields. Their respective complexes were synthesized by initial activation of the dimeric precursor [M(cym)Cl_2_]_2_ by sodium acetate, where M is either Ru(II) or Os(II). Addition of the appropriate 2-phenylbenzothiazole lead to the formation of the desired *C,N-*metallacycle. The reactions were performed either at 40°C (ruthenium complexes **1a−6a** and **1b**) or under microwave conditions (osmium complexes **2b−6b)**. After work-up the pure products were obtained by precipitation from dichloromethane (DCM) and *n*-hexane.

### Characterization

All ligands and complexes were characterized by means of ^1^H-NMR, ^13^C-NMR in DMSO-d_6_ (ligands) and CDCl_3_ (complexes), elemental analysis, and ESI-MS. ^1^H-^1^H-COSY, ^1^H-^13^C-HMBC, and ^1^H-^13^C-HSQC were recorded to unambiguously assign the detected NMR signals. Furthermore, their melting points and solubilities were determined. ^1^H and ^13^NMR spectroscopy confirmed the formation of the desired metallacycles. The ligand spectra showed two doublets for the 4-substituted phenyl ring (see Figures S1–S3). Upon *C,N*-coordination the symmetry is broken, yielding separate signals for the phenyl protons. Additionally, the coordinated benzothiazole ligand hinders inversion at the metal center in aprotic solvents such as CDCl_3_. As a consequence, the arene protons of *p*-cymene were found as four distinct doublets (see Figures S3–S9). At first glance complex spectra of **6a,b** look impure; however, it was found that partial protonation of the aniline took place due to common acid traces in CDCl_3_, leading to two sets of signals.

The crystal structure of **L5** and the ruthenium(II) and osmium(II) complex of 2-phenylbenzothiazole (**1a** and **1b**, respectively) was determined via X-ray diffraction analysis (Tables S2–S8).

**Figure 1:**
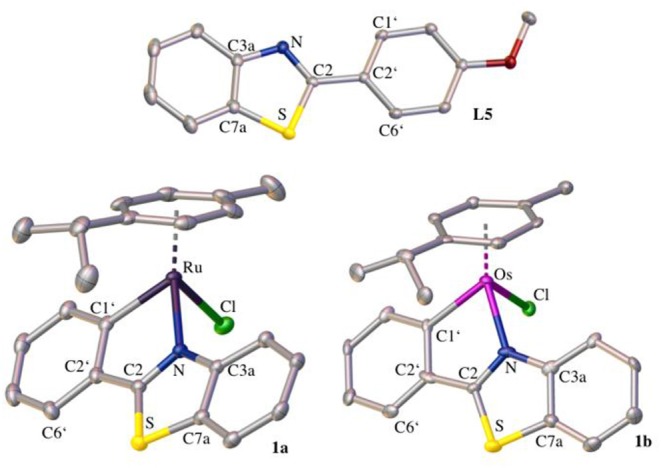
Crystal structures of **L5**, **1a**, and **1b** drawn at the 50% probability level. Hydrogen atoms are omitted for clarity.

The elemental cell of **L5** was composed of four molecules with slightly different bond lengths. **L5** crystallized in the monoclinic crystal system and in the crystal structure (Figure 1) the distortion of the thiazole ring due to the heteroatoms is clearly visible. The bond length between C3a and C7a ranges from 1.4072 to 1.4104 Å, whereas the carbon-nitrogen bonds are slightly shorter (C3a-N: 1.3858–1.3944 Å, C2-N: 1.3026–1.3113 Å) and the carbon-sulfur bonds are significantly longer (C7a-S: 1.7225–1.7341 Å, C2-S: 1.7566–1.7625 Å). This distortion is caused by the difference in size between the carbon atoms and the sulfur and nitrogen atoms. Further bond lengths and angles are listed in Table S1. Both the ruthenium and the osmium complex crystallized in an orthorhombic crystal system in the centrosymmetric space group P2_1_2_1_2_1._ The crystal structures (Figure 1) illustrate the typical piano-stool configuration, with the facial coordinated cym as the seat of the piano-stool and the chlorido ligand and the bidentate 2-phenylbenzothiazole ligand acting as the three legs. In the pseudo-octahedral geometry of the complex, a new distorted five-membered ring is formed by the chelating ligand and the metal center. The coordination bonds are very similar for both metal analogs, which was to be expected as the atomic radii for both metals differ only by 0.01 Å (Ru: 1.34 Å, Os: 1.35 Å). In the ruthenium complex, the bond length is 2.1115(19) Å and 2.047(2) Å for the coordination bond between the metal and the nitrogen and carbon atom, respectively. The analogous bond lengths in the osmium complex are 2.109(7) and 2.073(19) Å. The M–Cl distance is in the expected range, with 2.4133(6) and 2.411(2) Å in the ruthenium and osmium complex, respectively. Moreover, the coordination ring is not planar and features a torsion angle of 5.1(2)° (between M-N-C2-C1') in the ruthenium complex. In the osmium complex this twist is less pronounced with a torsion angle of 2.4(10)°. Due to this torsion, the thiazole ring loses its planarity as well and shows a slight torsion of 1.4(2)° in complex **1a** and 2.1(10)° in complex **1b** (between C3a-N-C2-S). When comparing the structure of the free ligand and the ligand in the complex, it is evident that the coordination to the metal atom causes the bond between the benzothiazole ring system and the phenyl ring as well as the bond C1'-C6' to shorten, while the bond C1'-C2' is elongated. Although this effect is apparent in both metal complexes, it is slightly more prominent in the osmium complex. In the benzothiazole ring system, more differences between the two complexes become apparent. For instance, in the ruthenium complex the bond C2-N is stretched minimally upon coordination (for the bond C3a-N no difference in bond length can be observed), whereas in the osmium complex both bonds C2-N and C3a-N appear shortened. In general, complexation with the osmium center seems to have a more pronounced effect on bond lengths and angles than coordination to ruthenium.

### Stability Investigations

The stability of **1a**, **6a**, **1b**, and **6b** in aqueous solution over a period of 48 h at 37°C was investigated to confirm sufficient stability for further biological experiments. All complexes form immediately the corresponding aqua species after dissolution in aqueous systems. Mass spectra were recorded after 1, 3, 6, 24, and 48 h. **L1** and **L6** were examined as well and were completely stable. As expected no solvent adducts or decomposition products were detected for the investigated organometallics over 48 h.

**1a** proved to be nearly stable over the entire period, with a characteristic [M-Cl]^+^ peak (*m/z* 446.07 ± 0.01, m_th_: 446.05), with only minor traces of a dimeric hydrolysis product starting to form after 6 h (*m/z* 550.06 ± 0.03), until it reached a total of 7% after 48 h. Complex **6a** showed the [M-Cl]^+^ peak (*m/z* 461.08 ± 0.01, m_th_: 461.07) and no solvent adduct peaks were found. Osmium complexes **1b** and **6b** were stable over the entire period and only the peaks [M-Cl]^+^ were found (*m/z* 536.11 ± 0.01, m_th_: 536.11) and (*m/z* 551.11 ± 0.01, m_th_: 551.12), respectively.

Understanding the affinity of the selected compounds (**1a**, **6a**, **1b**, and **6b**) toward small biomolecules like amino acids, proteins and nucleoside triphosphates gives valuable information about possible modes of action *in vivo*. In order to gain this understanding, the compounds were incubated with the respective biomolecule-containing solution for 48 h at 37°C. Again, the mass spectra were recorded after 1, 3, 6, 24, and 48 h. Solution 1 (equimolar mixture of l-cysteine [Cys], l-histidine [His] and l-methionine [Met] in 1% DMF) was used to point out preferences toward the typical donor amino acids. Solution 2 (equimolar mixture of *N*-Ac-l-cysteine [*N*-Ac-Cys], *N*-Ac-l-histidine [*N*-Ac-His], *N*-Ac-l-methionine [*N*-Ac-Met] and Se-methyl-l-cysteine [Cys-Se^Me^] in 1% DMF) was applied as an adapted amino acid model closer to proteins, where bidentate binding is not accessible. Solution 3 (equimolar mixture of Se-l-cystine and DTT in 1% DMF) was tested with regards to selenium binding affinity of the complexes. Solution 4 (equimolar mixture of 5′-ATP [ATP] and 5′-GTP [GTP] in 1% DMF) is a mixture used to determine possible binding preferences toward the typical DNA targets for metal-based anticancer agents. Solution 5 (equimolar mixture of solution 2 and solution 4) was employed for competitive observations and solution 6 is equimolar mixture of ubiquitin and cytochrome c as potential binding partners in biological systems.

When administered into the blood stream, the first biological nucleophiles able to interact with the compounds are serum proteins. To gain insight into preferences of the investigated complexes, they were incubated with different mixtures of biologically relevant amino acids and model proteins. When incubated with the amino acid containing solutions 1–3 and 5 all complexes showed the characteristic [M-Cl]^+^ peak [M(cym)(**L**)]^+^ (see Table S9). Additionally, all complexes formed a thermodynamically favored adduct with l-methionine with an amount of 49–62% of all assignable adducts with solution 1: [M(cym)(**L**)(Met)]^+^ (Ru, **L1**, 56%; Ru, **L6**, 49%; Os, **L1**, 56%; Os, **L6**, 62%; see Table S9). Complex **1a** also formed an additional methionine adduct lacking the ligand [Ru(cym)(Met)]^+^ (*m/z* 384.09 ± 0.02, m_th_: 384.06, 9%). When incubated with solution 2 all complexes formed a thermodynamically stable [M(cym)(**L**)(*N*-Ac-Met)]^+^ adduct in the range of 59–83% (Ru, **L1**, 59%; Ru, **L6**, 61%; Os, **L1**, 74%; Os, **L6**, 83%; see Table S9). The ruthenium complexes also formed several cysteine adducts: [Ru(cym)(**L**)(*N*-Ac-Cys)]^+^ (**L** = **L1**, *m/z* 607.04 ± 0.02, m_th_: 607.09, 7%; **L** = **L6**, *m/z* 624.07 ± 0.01, m_th_: 623.77, 11%) and [Ru(cym)(Cys-Se^Me^)]^+^ (*m/z* 417.99 ± 0.02, m_th_: 417.99, 5–9%). Complex **1a** also formed two additional l-cysteine adducts upon treatment with solution 2: [Ru(cym)_2_(*N*-Ac-Cys)_3_]^+^ (*m/z* 958.01 ± 0.01, m_th_: 958.12, 8%) and an adduct with *N*-acetylated l-cystine: [Ru(cym)_2_(*N*-Ac-Cys-Cys-*N*-Ac)]^+^ (*m/z* 795.04 ± 0.03, m_th_: 795.07, 3%). Apart from the characteristic [M-Cl]^+^, all complexes formed an adduct with DTT, rather than with Se-l-cysteine when incubated with solution 3: [M(cym)(**L**)(DTT)]^+^ (Ru, **L1**, 74%, Ru, **L6**, 55%; Os, **L1**, 63%; Os, **L6**, 52%; see Table S9). Overall, l-methionine was the preferred binding partner for all compounds, with the osmium complexes **1b** and **6b** exclusively forming the methionine adduct described above. The ruthenium complexes **1a** and **6a** also formed l-cysteine adducts in low amounts, especially **1a**. None of the complexes showed any affinity toward l-histidine. The selective DTT adduct formation over Se-l-cysteine allows the assumption that the mode of action of the investigated complexes does not depend on interference with selenium dependent pathways (relevant cell functions based on glutathione peroxidase, glutathione reductase and γ-glutamyl transpeptidase).

The reactivity of the complexes toward the proteins ubiquitin (Ub) and cytochrome c (Cyt) were investigated to gain better knowledge of their *in vivo* behavior. Cytochrome c has three possible binding sites (His26, His23, and Met65) and represents a possible biological target, while Ub has two possible binding sites (Met1 and His68) and was chosen as a model protein in order to understand the reactivity of the complexes to proteins in general. The solutions were diluted with 0.2% formic acid (v/v) prior to recording of the spectra, so the proteins occurred in their highest possible charged states (Ub: 7–14 and Cyt: 11–19). This convoluted the spectra for obvious reasons, hence the recorded mass spectra needed to be deconvoluted. In the following paragraph all percent values are percent of the assignable protein peaks, rather than percent of the assignable metal peaks.

When incubated with Cyt, none of the complexes showed any interaction and Cyt was only found in its unreacted form [Cyt] (*m/z* 12358.54 ± 0.42, m_th_: 12365), even though Met65 would be a possible binding site according to the amino acid experiments. In contrast, all complexes showed a mediocre reactivity toward Ub: [M(cym)(**L**)(Ub)]^+^ (Ru, **L1**, 12%; Ru, **L6**, 17%, Os, **L1**; 28%; Os, **L6**, 3%; see Table S9). Both ruthenium complexes also formed an adduct with Ub where the ligand was cleaved off: [Ru(cym)(Ub)]^+^ (*m/z* 8797.31 ± 0.66, m_th_: 8799.64, 9–11%). These results suggest that the reaction with cytochrome c is not a likely pathway for possible biological activity. On the other hand interactions with ubiquitin were observed even though the affinity was not very high.

The reactivity of all complexes toward the nucleoside-triphosphates ATP and GTP revealed a selectivity of the compounds toward guanine. The kinetically favored adduct was [M(cym)(**L**)(GTP)]^2−^ (Ru, **L1**, ~20–30%; Ru, **L6**, ~60%; Os, **L1**, ~74%; Os, **L6**, ~70%; see Table S9). Complexes **1a** and **6a** also formed other kinetically favored products: [Ru(cym)(GTP)(DMF)]^2−^ (*m/z* 415.95 ± 0.02, m_th_: 416.03, ~30–40%, only **1a**) and [(Ru(cym)(GTP)]^−^ (*m/z* 757.95 ± 0.01, m_th_: 757.45, 26–60%). The thermodynamically stable products of ruthenium complexes **1a** and **6a** were [Ru(cym)(GTP)]^2−^ (*m/z* 377.49 ± 0.01, m_th_: 377.50, 36–48%) and an adduct with ATP [Ru(cym)(ATP)]^−^ (*m/z* 739.96 ± 0.01, m_th_: 740.01, 27–39%). Complex **6b** also formed a second guanine adduct: [(Os(cym)(DMF)_2_(GTP)]^−^ (*m/z* 992.02 ± 0.02, m_th_: 992.16, ~30% during the entire period). The osmium complex **1b** also showed reactivity with adenine: [(Os(cym)(DMF)_2_(ATP)]^−^ (*m/z* 977.02 ± 0.02, m_th_: 977.17, ~26% during the entire period). These results suggest that all complexes have at least twice the reactivity toward guanine than to adenine.

Incubation with the mixture of amino acids and nucleotides (solution 5) revealed that all complexes showed the same distribution of amino acid adducts as described for incubation with solution 2, and none with the nucleotides. These results suggest that biological activity can be primarily attributed to protein interactions, whereas DNA as the biological target is rather unlikely.

### Antiproliferative Activity in Cancer Cells

The antiproliferative activity of ligands **L1–L6** and the respective ruthenium (**1a−6a**) and osmium (**1b−6b**) complexes was evaluated in human ovarian teratocarcinoma (CH1/PA-1), colon (SW480) and non-small cell lung carcinoma (A549) cell lines (Table 1, Figures S10–S12). In general, the free ligands hardly show relevant antiproliferative effects in the tested cell lines. Overall coordination of the benzothiazole ligand scaffold to organometallic fragments resulted in highly cytotoxic compounds with IC_50_ values in the low micromolar range. Based on the obtained data most complexes were at least about one order of magnitude more active than the free ligand. Although CH1/PA-1 cells were found to be the most sensitive of the cell lines used, the difference in efficacy compared to the more chemo-resistant A549 and SW480 cells is remarkably small. The antiproliferative potency of the investigated compounds, hence, seems to depend on the metal center and increases in the following order: ligands < < ruthenium(II) compounds < osmium(II) compounds; with differences in cytotoxicity of the complexes being far less pronounced. The obtained trends are in good agreement with recently reported triazole-based metallacycles, where also a pronounced increase of cytotoxicity upon *C,N*-coordination to organoruthenium and –osmium moieties was observed (Riedl et al., 2018). With regard to variation of the substituent on the phenylbenzothiazole ligand, consistent structure-activity relationships are difficult to derive, as their impact on cytotoxic potency is rather small. For further biological experiments we have chosen ruthenium complexes **2a**, **6a** and osmium derivatives **2b**, **6b** to cover the whole range of determined IC_50_ values.

**Table 1:** Inhibition of cancer cell growth in three human cancer cell lines; 50% inhibitory concentrations (μM; means ± standard deviations), obtained by the MTT assay (exposure time: 96 h).

**Compound**	**A549**	**SW480**	**CH1/PA-1**
**L1**	>40	>40	>40
**L2**	>50	>50	>50
**L3**	>50	>50	>50
**L4**	>100	>100	>100
**L5**	76 ± 8	>100	44 ± 7
**L6**	78 ± 8	143 ± 8	27 ± 4
**1a**	12 ± 1	6.8 ± 0.9	4.1 ± 0.3
**2a**	8.4 ± 0.4	4.0 ± 0.3	2.7 ± 0.3
**3a**	8.2 ± 0.6	4.1 ± 0.2	4.0 ± 0.4
**4a**	14 ± 2	8.5 ± 0.7	7.8 ± 0.7
**5a**	10.5 ± 0.2	5.3 ± 0.7	4.2 ± 0.6
**6a**	16 ± 2	13 ± 1	6.4 ± 1.5
**1b**	4.0 ± 0.6	4.8 ± 0.9	1.2 ± 0.2
**2b**	4.0 ± 0.7	4.4 ± 0.1	2.1 ± 0.3
**3b**	4.9 ± 1.0	5.5 ± 1.1	2.0 ± 0.3
**4b**	5.6 ± 1.3	6.1 ± 1.5	1.7 ± 0.1
**5b**	3.9 ± 0.6	5.0 ± 1.0	1.3 ± 0.2
**6b**	8.7 ± 0.7	7.2 ± 1.6	2.5 ± 0.5

### Apoptosis Assay

Flow-cytometric analysis of SW480 cells treated with **2a**, **2b**, **6a**, and **6b** for 24 h and then double-stained with an annexin V-FITC conjugate (AV) and propidium iodide (PI) revealed strong apoptotic effects of the Ru compound **2a** (Figure S13), comparable to the positive control (50 μM of Pt complex KP1998; Scaffidi-Domianello et al., 2012). Surprisingly, the Os analog **2b**, which is as potent as **2a** according to the 96-h MTT assay, does not induce apoptosis within 24 h (Table S10). Compound **6a**, the least potent of the Ru series according to the MTT assay, induces apoptosis to a similar extent as **2a** at 20 μM, but is by far surpassed by the latter at 40 μM (18% vs. 88% of apoptotic cells in total). The Os analog **6b** is less active than both of them, but induces apoptosis noticeably (14%) at 40 μM.

### Cellular Accumulation

Cellular accumulation of these compounds in SW480 cells after 2 h exposure is generally high with rather small differences: **6b** (382 ± 86 fg Os/cell), **2a** (301 ± 86 fg Ru/cell), **6a** (254 ± 58 fg Ru/cell), **2b** (243 ± 83 fg Os/cell). Since this rank order does neither correlate with antiproliferative nor with apoptotic potency, the high initial uptake alone cannot account for the outstanding effects of compound **2a** in the apoptosis assay.

### Colocalization of L6 and 6a With Lysosomes

**Figure 2:**
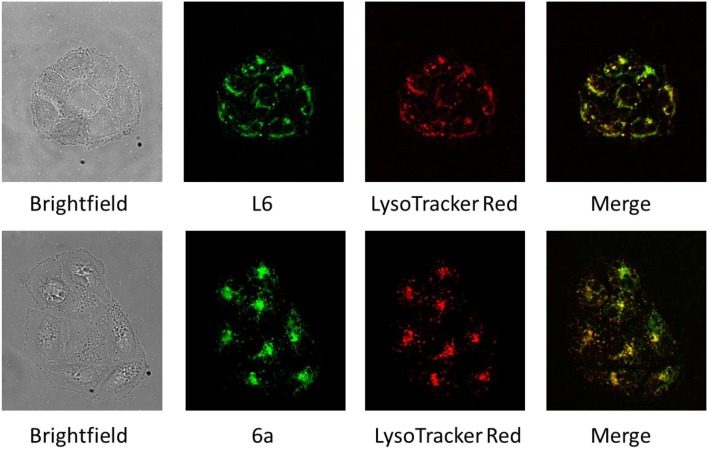
Brightfield and fluorescence microscopy images of SW480 cells treated with **L6** and **6a** (green), stained with LysoTracker Red (red), and merged images revealing an extensive colocalization of fluorescence (yellow).

The fluorescence properties of **L6** and **6a** were exploited to visualize their intracellular distribution in SW480 colon carcinoma cells by fluorescence microscopy in a live cell setting (Figure 2). Staining with LysoTracker Red was used to study the localization of both compounds with regard to lysosomal compartments, and a high degree of overlap between the fluorescence images strongly suggests a pronounced colocalization with lysosomes (see Figure 2). To statistically confirm the visual colocalization of the drug with the acidic compartments of the cell (Figure 2), correlation analysis was conducted. Pearson's coefficients of 0.77 ± 0.08 and 0.81 ± 0.06 between red fluorescence (LysoTracker Red) and green fluorescence (corresponding to compounds **L6** and **6a**, respectively) were obtained, indicating a substantial extent of correlation. Lysosomes might represent a potential target for the novel Ru complexes with alkaline and lipophilic physiochemical properties. Trapping of the compounds in acidic compartments due to changes in charge based on the weakly basic properties of the aniline group is conceivable, but remains speculative at this stage. Furthermore, morphological differences between the samples treated with two different compounds (**L6** and **6a**) can be observed. In comparison to cells treated with **L6**, the cells exposed to **6a** show a pronounced cytoplasmic disparity due to beginning vacuolation.

## Conclusions

A series of 2-phenylbenzothiazoles and their corresponding ruthenium(II) and osmium(II) half-sandwich complexes were synthesized. The obtained compounds were characterized by standard analytical methods and their aqueous stability was investigated. Additionally, ESI-MS studies showed general binding preferences with regards to possible binding partners, after administration. Surprisingly, no complex showed any affinity toward *Se*- and *N*-donor amino acids. Almost only sulfur-donor amino acid adducts were found, especially with thioether moieties over thiols; a preference also observed with 5'-GTP adducts, which were almost exclusively formed, leaving present 5'-ATP untouched. However, competition experiments revealed a pronounced affinity toward amino acids over NTPs. Furthermore, the IC_50_ values were determined in human cancer cell lines, and proved to be in the low μM range, with comparatively small differences between the most sensitive and the most resistant cell line. Although osmium complexes showed a stronger overall effect on cancer cell proliferation over 96 h than their ruthenium counterparts, the ruthenium complexes more potently induced apoptosis within 24 h. For one selected ligand and its ruthenium complex, fluorescence microscopic images suggest a high accumulation in lysosomes and other subcellular acidic compartments. The cellular accumulation of the investigated complexes was in a very similar range for all tested organometallics and could hence not provide an explanation for the different cytotoxic properties of the ruthenium and osmium metallacycles.

## Data Availability Statement

All datasets generated for this study are included in the article/Supplementary material.

## Author Contributions

WK and BK: conceptualization and methodology. MJ, AR, SM-M, WK, and BK: validation. SM, HG, KS, MK, AR, and MH: formal analysis. SM, KC, HG, MK, AR, and MH: investigation. AR, SM-M, MJ, WK, and BK: resources and data curation. SM, HG, MK, MJ, and WK: writing—original draft preparation. SM, HG, and WK: visualization. WK and BK: supervision, project administration, and funding acquisition.

### Conflict of Interest

The authors declare that the research was conducted in the absence of any commercial or financial relationships that could be construed as a potential conflict of interest.
