# Supplementary material for: Investigations on the Anticancer Potential of Benzothiazole-Based Metallacycles
Source: Front Chem. 2020 Apr 3;8:209. doi: 10.3389/fchem.2020.00209 (PMC7147246; doi:10.3389/fchem.2020.00209)
Supplement: Supplementary file 1 [file Data_Sheet_1.pdf]

Supplementary Material

Investigations on the Anticancer Potential of Benzothiazole-Based Metallacycles

S. Mokesch[1$], K. Cseh[1$], H. Geisler[1], M. Hejl[1], M. H. M. Klose[1], A. Roller[1], S. M. Meier-Menches[2,3], M. A. Jakupec[1,2], W. Kandioller[*1,2] and B. K. Keppler[1,2]

[1]Institute of Inorganic Chemistry, Faculty of Chemistry, University of Vienna, Vienna, Austria

[2]Research Cluster "Translational Cancer Therapy Research, University of Vienna, Vienna, Austria

[3]Department of Analytical Chemistry, Faculty of Chemistry, University of Vienna, Vienna, Austria

*** Correspondence:**
Dr. Wolfgang Kandioller
wolfgang.kandioller@univie.ac.at

[$]Both authors contributed equally to this work

Contents

1 ^{1}H NMR spectra 2

2 Crystallographic Data 11

3 Biological data 16

3.1 FACS Studies 20

3.2 Cellular Uptake Studies 22

4 References 22

1 ^{1}H NMR spectra

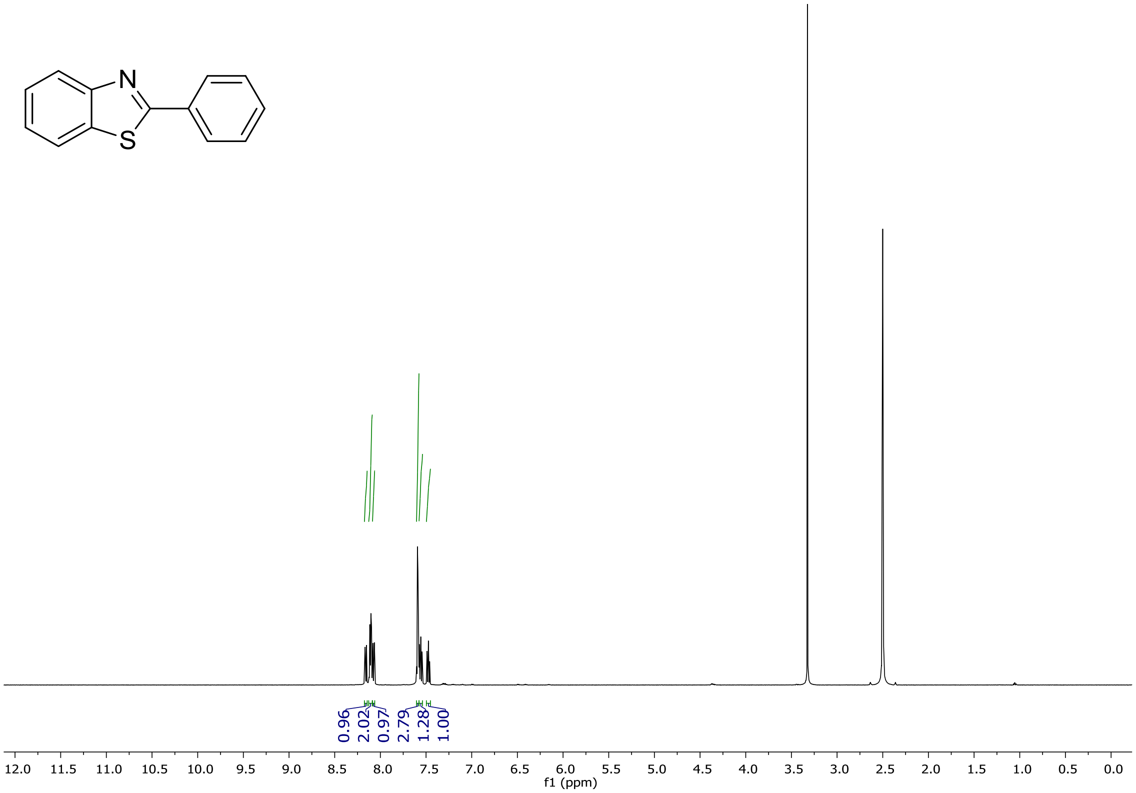

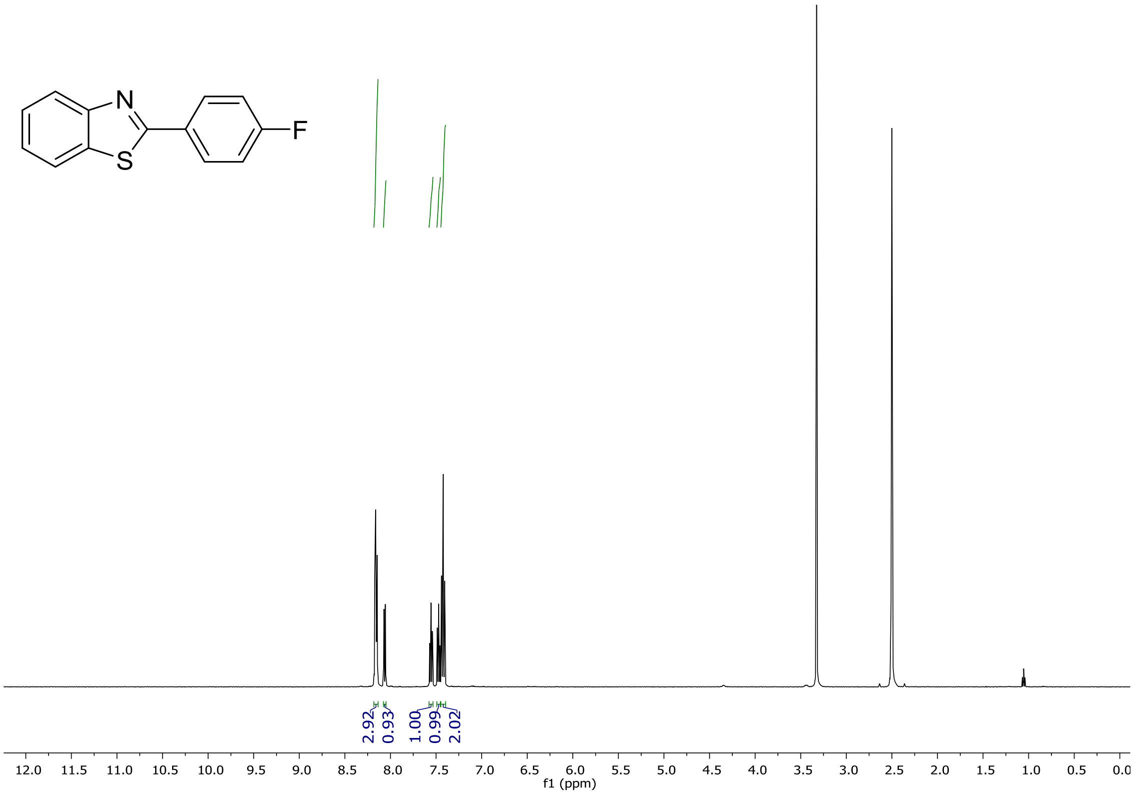

Figure S1: ^{1}H-NMR spectra of compound L1 (top) and L2 (bottom)

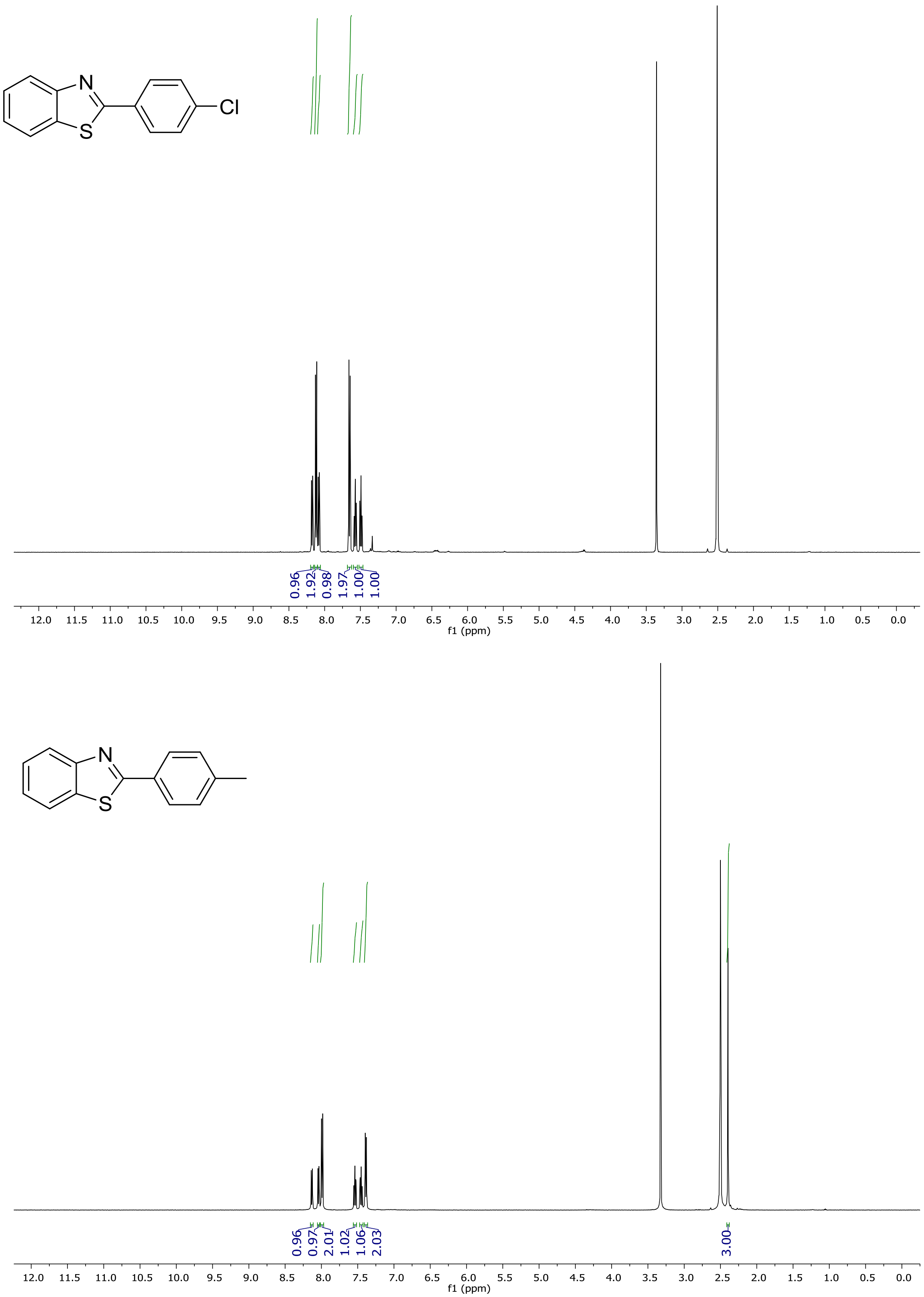

Figure S2: ^{1}H-spectra of compound L3 (top) and L4 (bottom)

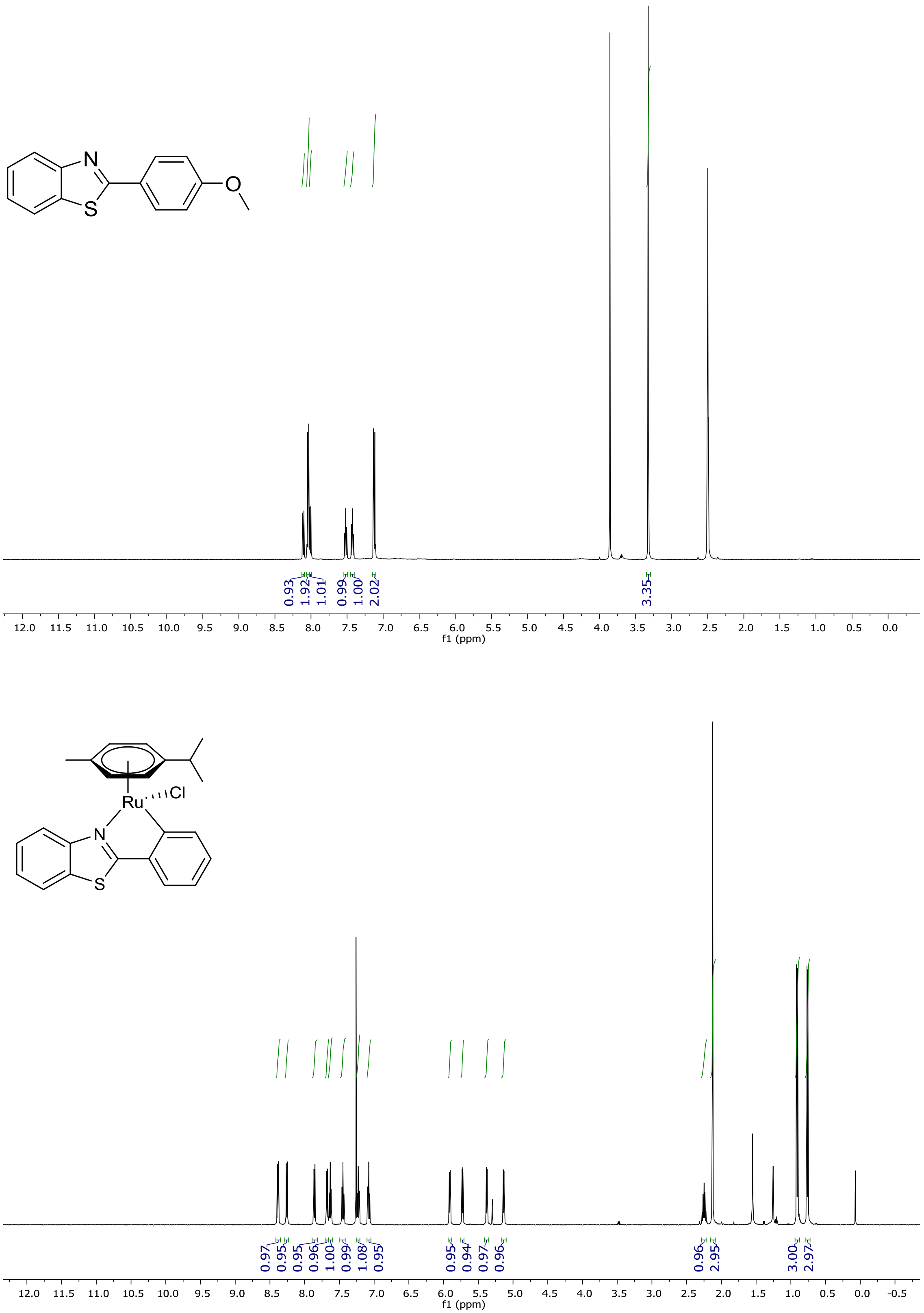

Figure S3: ^{1}H-spectra of compound L5 (top) and 1a (bottom)

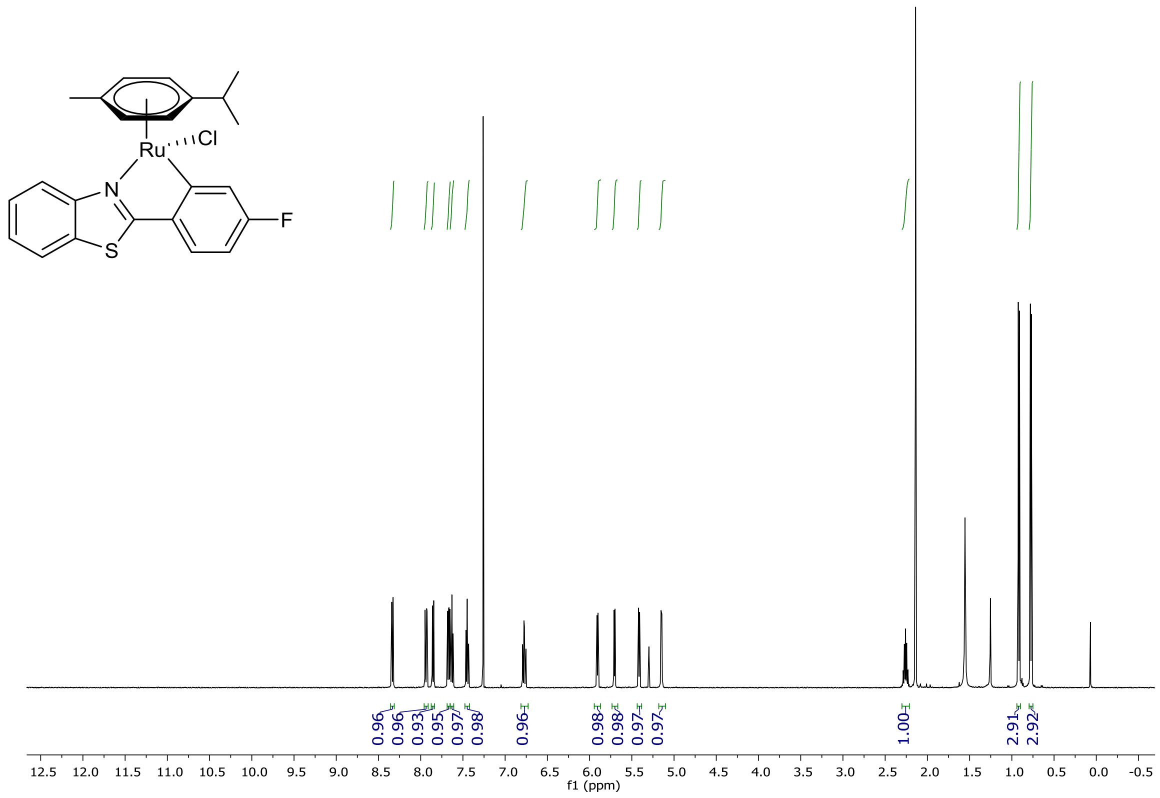

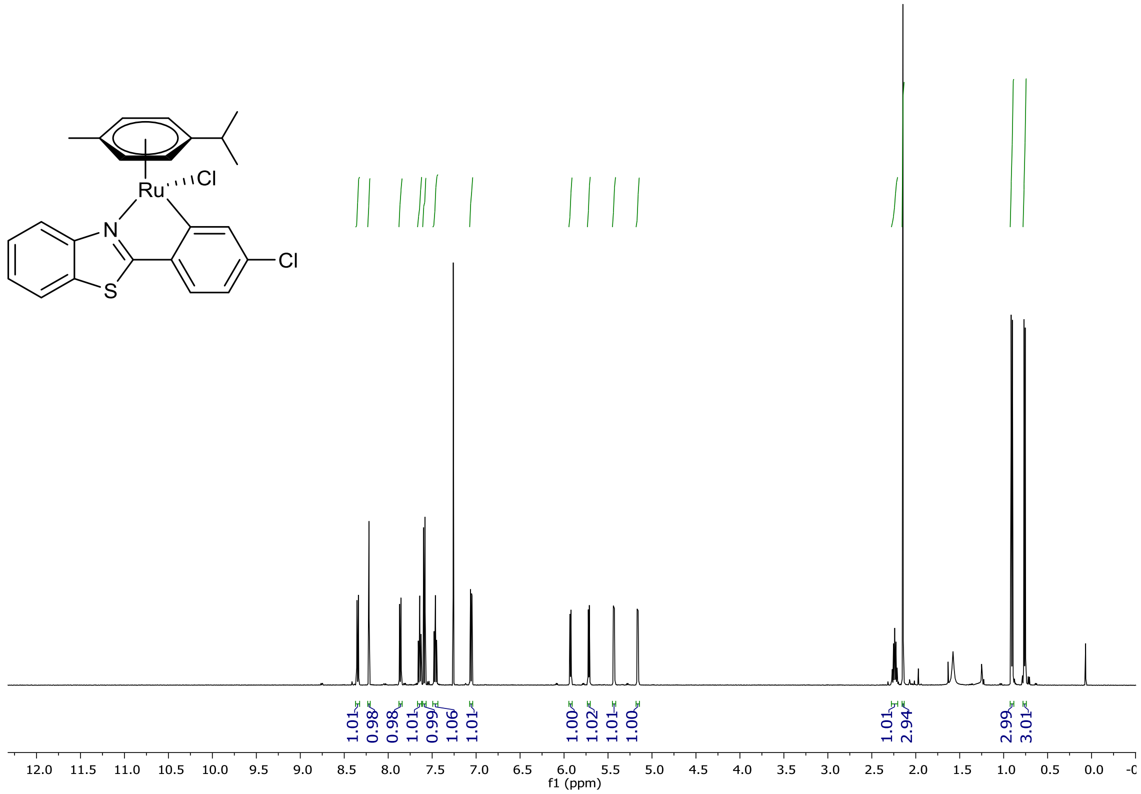

Figure S4: ^{1}H-spectra of compound 2a (top) and 3a (bottom)

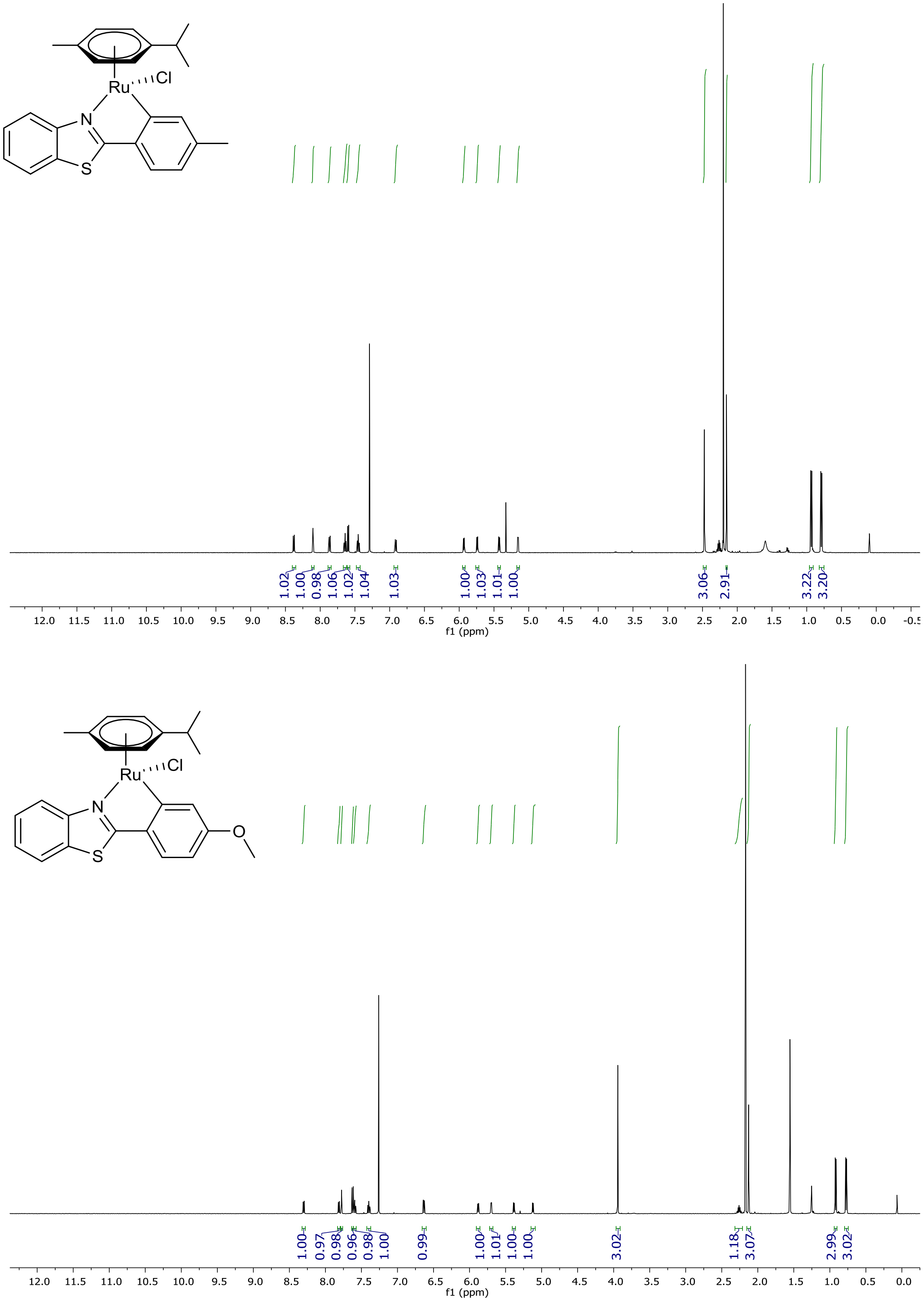

Figure S5: ^{1}H-spectra of compound 4a (top) and 5a (bottom)

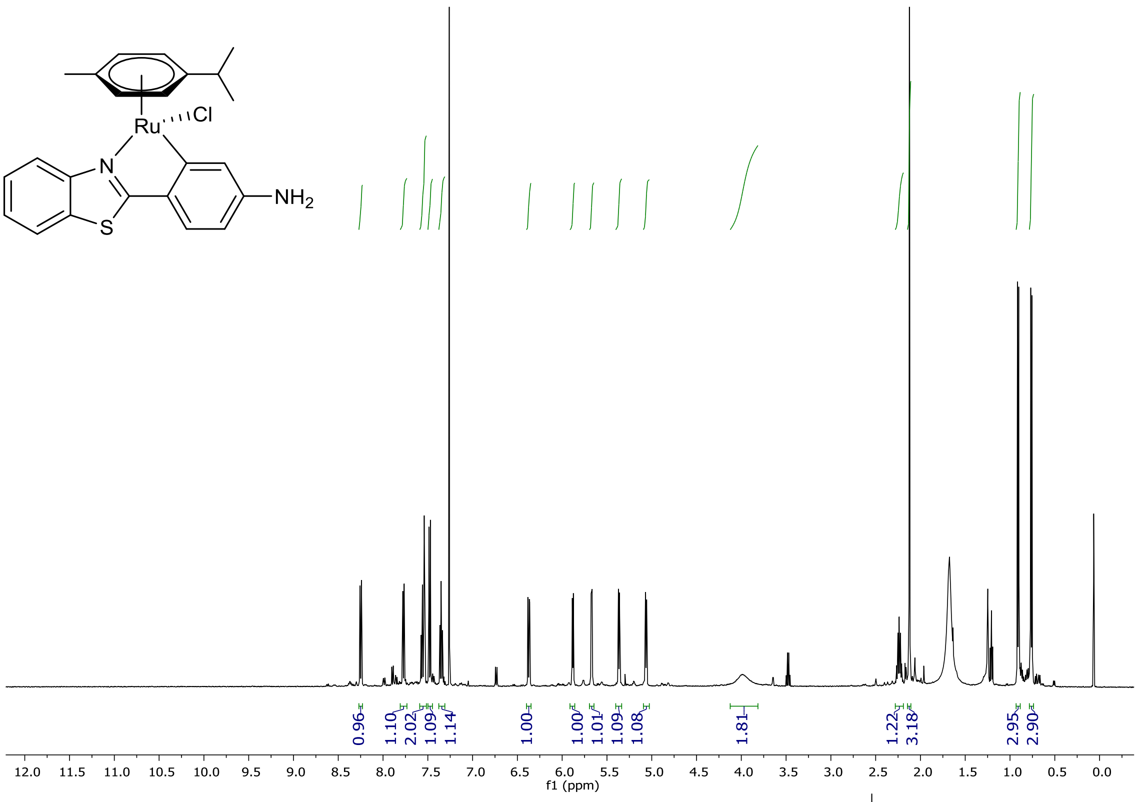

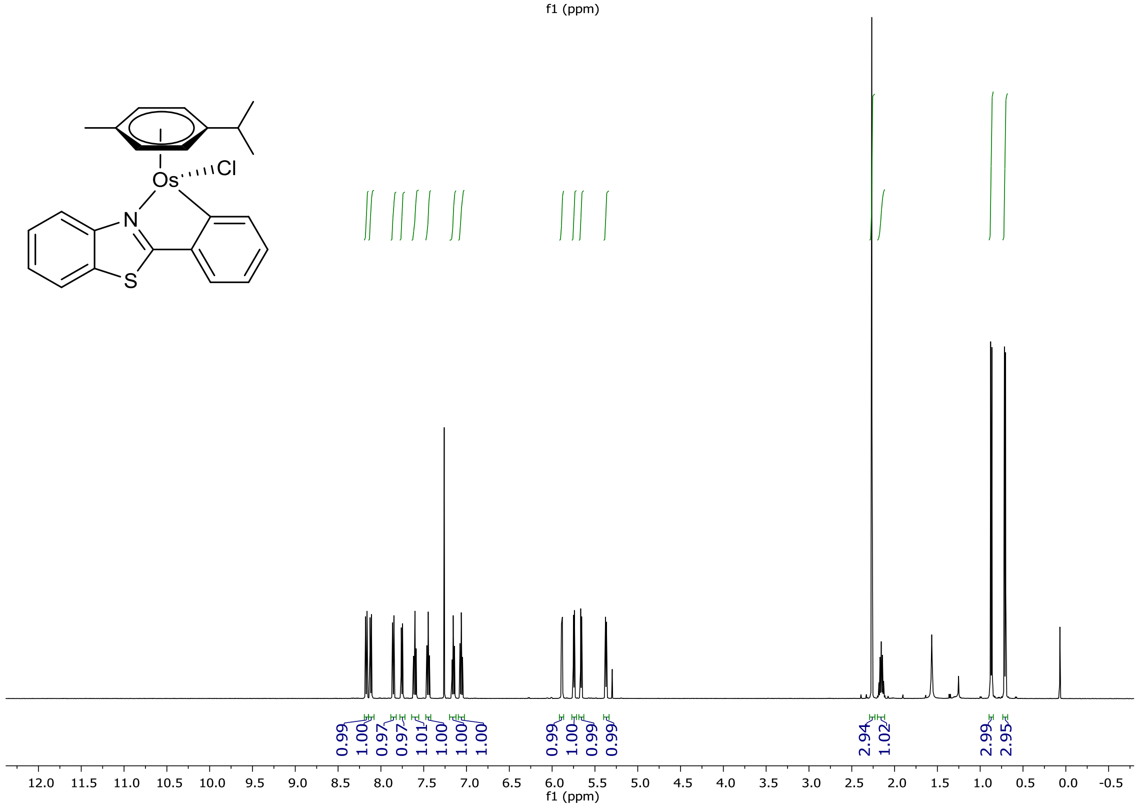

Figure S6: ^{1}H-spectra of compound 6a (top) and 1b (bottom)

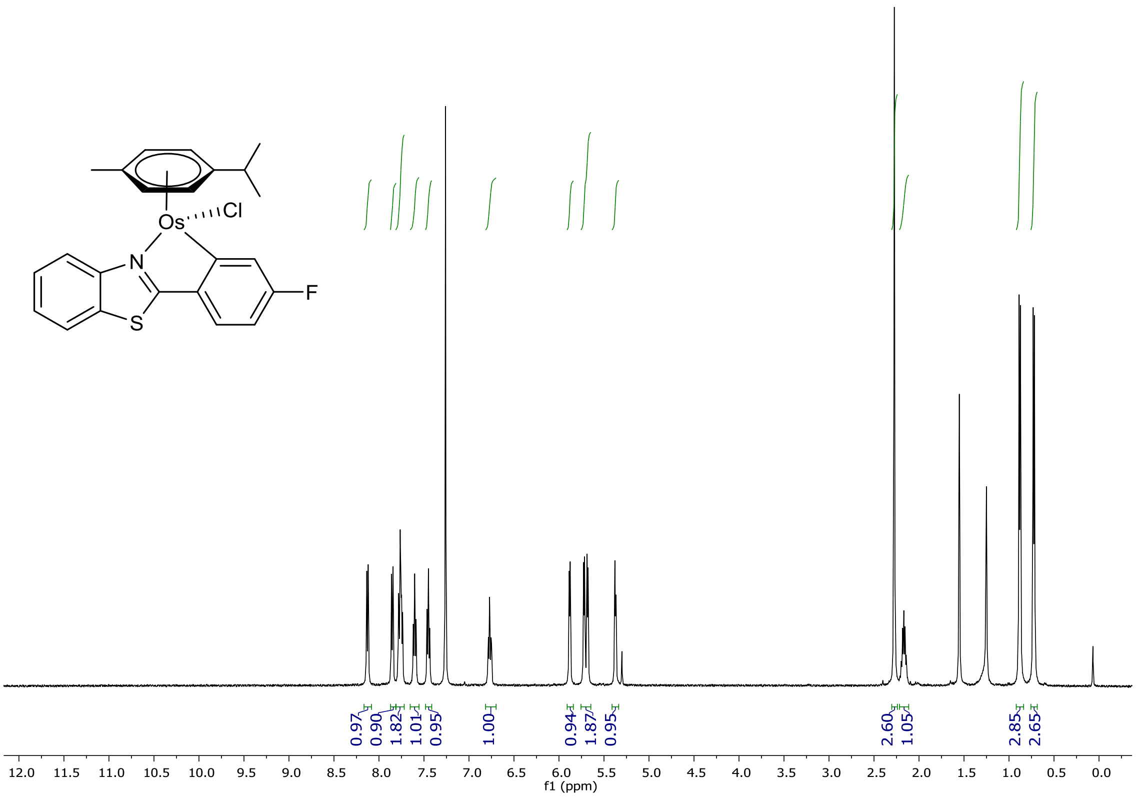

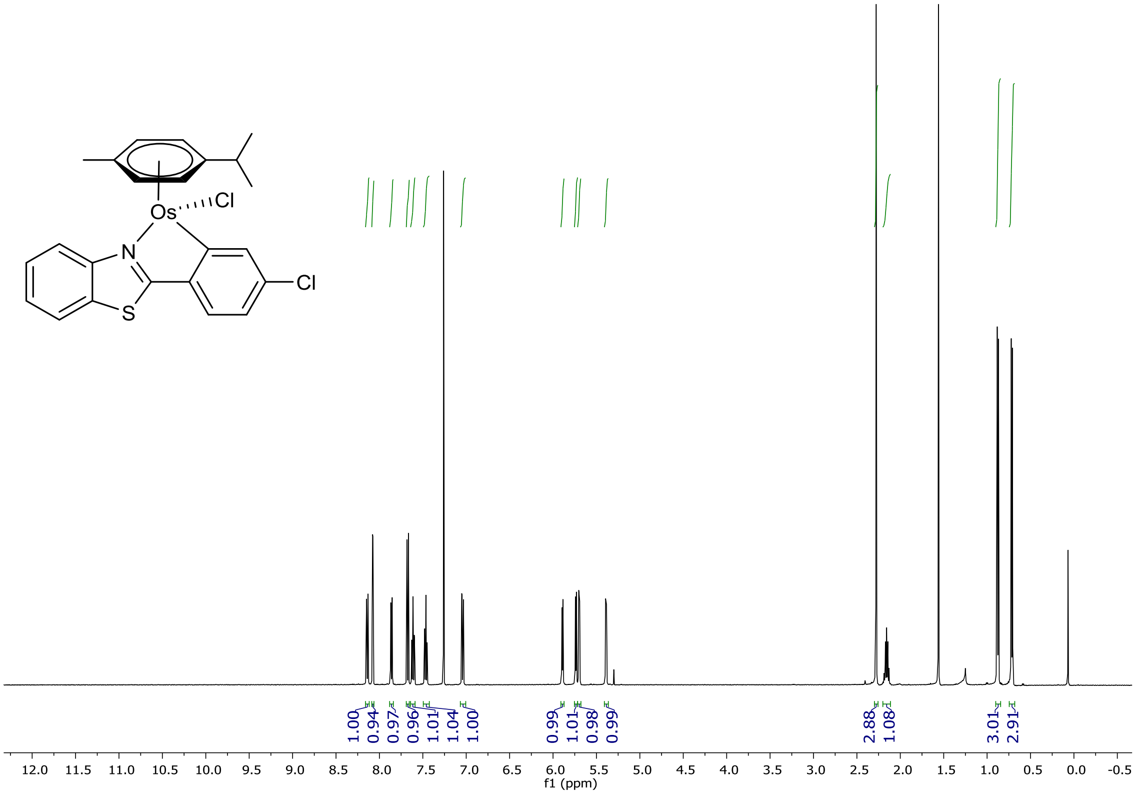

Figure S7: ^{1}H-spectra of compound 2b (top) and 3b (bottom)

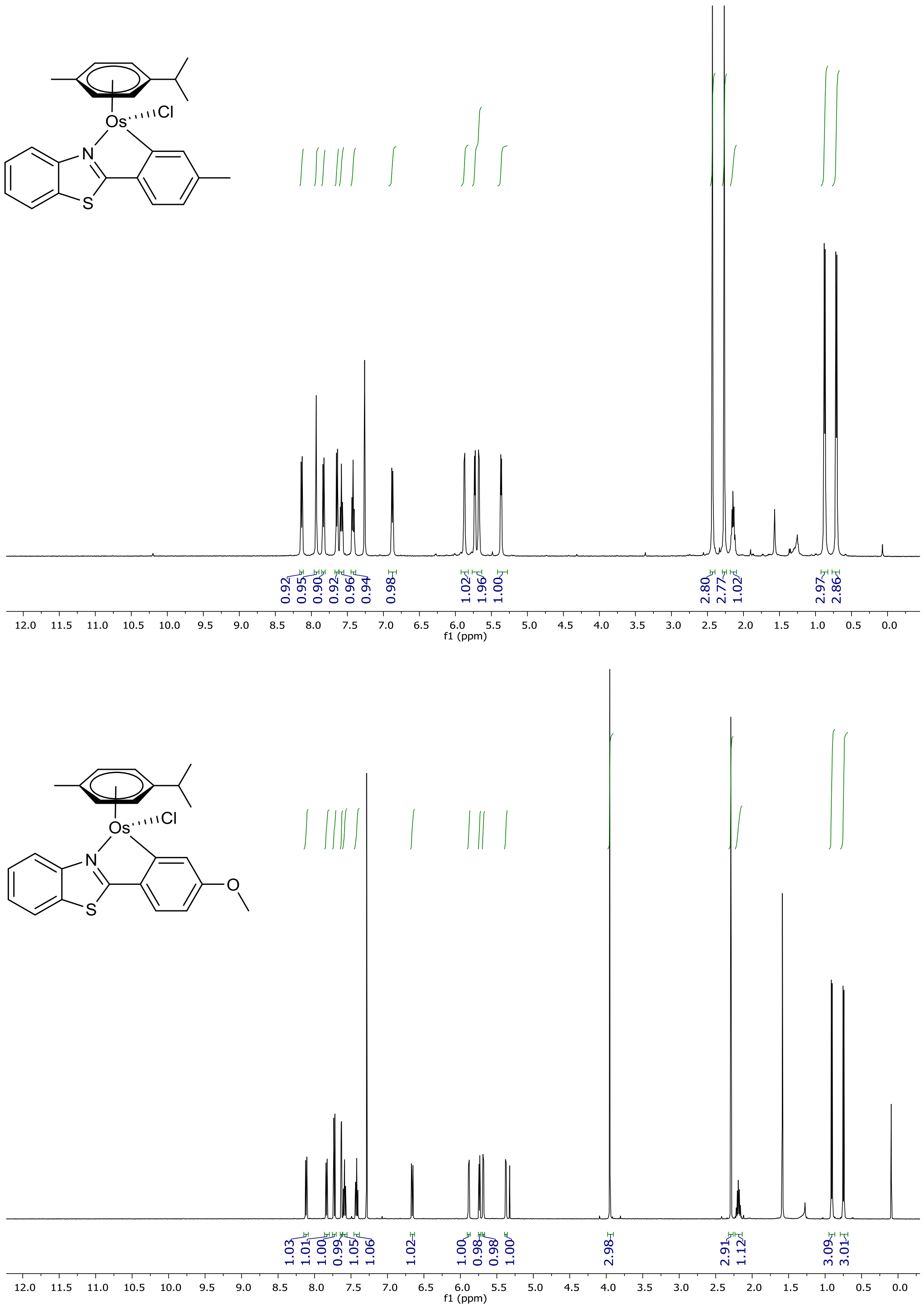

Figure S8. ^{1}**H-spectra of compound 4b (top) and 5b (bottom)**

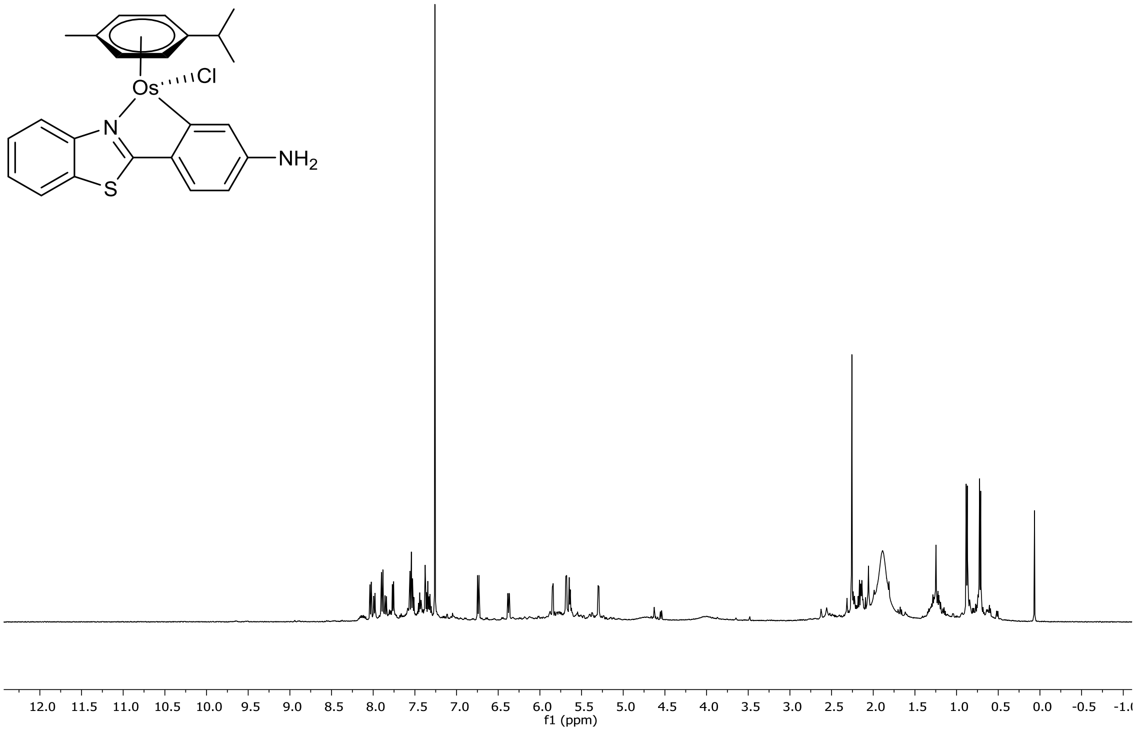

Figure S9: ^{1}H-spectrum of compound 6b

2 Crystallographic Data

Table S1. Experimental parameters and CCDC-Codes

**Short**	**Machine**	**Source**	**Temp.**	**Detector Distance**	**Time/ Frame**	**#Frames**	**Frame width**	**CCDC**
	Bruker		[K]	[mm]	[s]		[°]	
**1a**	D8	Mo	100	35	16	2954	0.4	1978569
**1b**	X8	Mo	100	35	5	440	0.5	1978570
**L5**	D8	Mo	100	34	18	2326	0.5	1978571

Table S2. Relevant bond lengths, angles and torsion angles in compounds 5. 1a. and 1b

Compound	**5**	**1a**	**1b**
M-Cl [Å]	-	2.4133(6)	2.411(2)
M-N [Å]	-	2.1115(19)	2.109(7)
M-C2’ [Å]	-	2.047(2)	2.073(10)
M-Cym_{centr}	-	1.712	1.704
C2-N [Å]	1.3026-1.3113	1.318(3)	1.301(12)
C3a-N [Å]	1.3858-1.3944	1.392(3)	1.374(14)
C2-S [Å]	1.7566-1.7625	1.732(2)	1.733(8)
C7a-S [Å]	1.7225-1.7341	1.747(3)	1.746(11)
C3a-C7a [Å]	1.4072-1.4104	1.400(3)	1.427(13)
C2-C1’ [Å]	1.4637-1.4662	1.444(3)	1.439(15)
C1’-C2’ [Å]	1.3942-1.3979	1.411(3)	1.425(13)
C1’-C6’ [Å]	1.4056-1.4108	1.396(4)	1.389(14)
N-M-C2’ [°]	-	77.52(8)	76.6(4)

Cl-M-N [°]	-	88.93(5)	83.9(2)
Cl-M-C2’ [°]	-	84.93(6)	86.4(2)
C2-N-M [°]	-	115.00(15)	116.4(7)
C1’-C2’-M [°]	-	116.84(15)	116.4(8)
C2-C1’-C2’ [°]	119.25-119.83	112.25(19)	111.7(9)
C3a-N-C2 [°]	109.73-110.76	111.49(19)	112.8(8)
N-C2-S [°]	115.43-115.87	115.29(16)	114.8(8)
C2-S-C7a [°]	89.14-89.25	89.37(10)	90.1(5)
C2’-C1’-C6’ [°]	118.59-118.72	123.2(2)	123.3(11)
M-N-C2-C1’ [°]	-	5.1(2)	2.4(10)
C3a-N-C2-S [°]	-	1.4(2)	2.1(10)

Table S3. Sample and crystal data of L5

**Chemical formula**	C14H11NOS	**Crystal system**	monoclinic	
**Formula weight [g/mol]**	241.3	**Space group**	*C2/c*	
**Temperature [K]**	100	**Z**	32	
**Measurement method**	\Φ and \ω scans	**Volume [Å^3]**	9182.1(7)	
**Radiation (Wavelength [Å])**	MoKα (λ = 0.71073)	**Unit cell dimensions [Å] and [°]**	38.449(2)	90
**Crystal size [mm^3]**	0.63 × 0.181 × 0.1		9.6018(3)	103.666(3)
**Crystal habit**	clear yellowblock		25.5966(10)	90
**Density (calculated) [g/cm^3]**	1.396	**Absorption coeff. / [mm^{-1}]**	0.262	
**Abs. correction Tmin**	0.6055	**Abs. correction Tmax**	0.7460	
**Abs. correction type**	multiscan	**F(000) [e$^-$]**	4032	

Table S4. Data collection and structure refinement of L5

**Index ranges**	$-54 \le h \le 54$. $-13 \le k \le 13$. $-36 \le l \le 36$	**Theta range for data collection [°]**	4.586 to 60.242	
**Reflections number**	227554	**Data / restraints / parameters**	13526/0/617	
**Refinement method**	Least squares	**Final R indices**	all data	R1 = 0.0526. wR2 = 0.1134
**Function minimized**	$\Sigma\ w(F_o^2 - F_c^2)^2$		I>2σ(I)	R1 = 0.0383. wR2 = 0.1030
**Goodness-of-fit on F^2**	1.013	**Weighting scheme**	$w=1/[\sigma^2(F_o^2)+(0.0588P)^2+9.5500P]$	
**Largest diff. peak and hole [e Å^{-3}]**	0.91/-0.32		where $P=(F_o^2+2F_c^2)/3$	

Table S5. Sample and crystal data of 1a

**Chemical formula**	C23H22ClNRuS	**Crystal system**	orthorhombic	
**Formula weight [g/mol]**	480.99	**Space group**	*Pna21*	
**Temperature [K]**	100	**Z**	4	
**Measurement method**	\Φ and \ω scans	**Volume [Å^3]**	1956.05(16)	
**Radiation (Wavelength [Å])**	MoKα (λ = 0.71073)	**Unit cell dimensions [Å] and [°]**	7.9904(4)	90
**Crystal size [mm^3]**	0.183 × 0.173 × 0.099		14.5823(7)	90
**Crystal habit**	clear orange block		16.7875(8)	90
**Density (calculated) [g/cm^3]**	1.633	**Absorption coeff. / [mm^{-1}]**	1.053	
**Abs. correction Tmin**	0.6927	**Abs. correction Tmax**	0.7460	
**Abs. correction type**	multiscan	**F(000) [e$^-$]**	976	

Table S6. Data collection and structure refinement of 1a

**Index ranges**	$-11 \le h \le 11$. $-20 \le k \le 20$. $-23 \le l \le 23$	**Theta range for data collection [°]**	3.7 to 60.198	
**Reflections number**	96508	**Data / restraints / parameters**	5762/1/247	
**Refinement method**	Least squares	**Final R indices**	all data	R1 = 0.0184. wR2 = 0.0465
**Function minimized**	$\Sigma\ w(F_o^2 - F_c^2)^2$		I>2σ(I)	R1 = 0.0180. wR2 = 0.0461
**Goodness-of-fit on F^2**	1.1	**Weighting scheme**	$w=1/[\sigma^2(F_o^2)+(0.0258P)^2+0.8970P]$	
**Largest diff. peak and hole [e Å^{-3}]**	0.98/-0.58		where $P=(F_o^2+2F_c^2)/3$	

Table S7. Sample and crystal data of 1b

**Chemical formula**	C23H22ClNOsS	**Crystal system**	orthorhombic	
**Formula weight [g/mol]**	570.12	**Space group**	*P212121*	
**Temperature [K]**	100	**Z**	4	
**Measurement method**	\Φ and \ω scans	**Volume [Å^3]**	1941.52(13)	
**Radiation (Wavelength [Å])**	MoKα (λ = 0.71073)	**Unit cell dimensions [Å] and [°]**	7.6690(3)	90
**Crystal size [mm^3]**	0.160 × 0.150 × 0.050		10.4181(3)	90
**Crystal habit**	clear orange block		24.3005(11)	90
**Density (calculated) [g/cm^3]**	1.95	**Absorption coeff. / [mm^{-1}]**	6.821	
**Abs. correction Tmin**	0.4696	**Abs. correction Tmax**	0.7460	
**Abs. correction type**	multiscan	**F(000) [e$^-$]**	1104	

Table S8. Data collection and structure refinement of 1b

**Index ranges**	$-9 \le h \le 8$. $-12 \le k \le 12$. $-29 \le l \le 24$	**Theta range for data collection [°]**	5.15 to 50.69	
**Reflections number**	7981	**Data / restraints / parameters**	3562/18/247	
**Refinement method**	Least squares	**Final R indices**	all data	R1 = 0.0352. wR2 = 0.0660
**Function minimized**	Σ w(F_o^2 - F_c^2)^2		I>2σ(I)	R1 = 0.0320. wR2 = 0.0643
**Goodness-of-fit on F^2**	1.055	**Weighting scheme**	w=1/[$\sigma^2(F_o^2)+(0.0235P)^2+$ 3.1903P]	
**Largest diff. peak and hole [e Å^{-3}]**	2.55/-1.18		where P=(F_o^2+2F_c^2)/3	

3 Abundant adducts found in different ESI-mass spectra

Table S9. Common adducts and their measured and theoretical m/z ratios

	m/z		
adduct	found	st dev	calc
[**1a**-Cl]$^+$	446.07	0.01	446.05
[**6a**-Cl]$^+$	461.08	0.01	461.07
[**1b**-Cl]$^+$	536.11	0.01	536.11
[**6b**-Cl]$^+$	551.12	0.01	551.12
[Ru(cym)(**L1**)(Met)]$^+$	595.07	0.01	595.11
[Ru(cym)(**L6**)(Met)]$^+$	610.08	0.01	610.12
[Os(cym)(**L1**)(Met)]$^+$	685.12	0.01	685.17
[Os(cym)(**L6**)(Met)]$^+$	700.13	0.01	700.18
[Ru(cym)(**L1**)(*N*-Ac-Met)]$^+$	637.08	0.01	637.12
[Ru(cym)(**L6**)(*N*-Ac-Met)]$^+$	652.09	0.01	652.13
[Os(cym)(**L1**)(*N*-Ac-Met)]$^+$	727.11	0.01	727.18
[Os(cym)(**L6**)(*N*-Ac-Met)]$^+$	742.11	0.01	742.19
[Ru(cym)(**L1**)(DDT)]$^+$	598.00	0.01	598.07
[Ru(cym)(**L6**)(DTT)]$^+$	613.02	0.01	613.08
[Os(cym)(**L1**)(DDT)]$^+$	688.05	0.01	688.13
[Os(cym)(**L6**)(DDT)]$^+$	703.07	0.01	703.14
[Ru(cym)(**L1**)(Ub)]$^+$	9008.3	1.3	9009.69
[Ru(cym)(**L6**)(Ub)]$^+$	9023.46	0.42	9024.70
[Os(cym)(**L1**)(Ub)]$^+$	9103.25	8.13	9100.75
[Os(cym)(**L6**)(Ub)]$^+$	9113.42	1.73	9114.75
[Ru(cym)(**L1**)(GTP)]$^{2-}$	483.00	0.02	483.02
[Ru(cym)(**L6**)(GTP)]$^{2-}$	490.51	0.01	490.53
[Os(cym)(**L1**)(GTP)]$^{2-}$	528.00	0.01	528.05
[Os(cym)(**L6**)(GTP)]$^{2-}$	535.98	0.02	536.06

4 Biological data

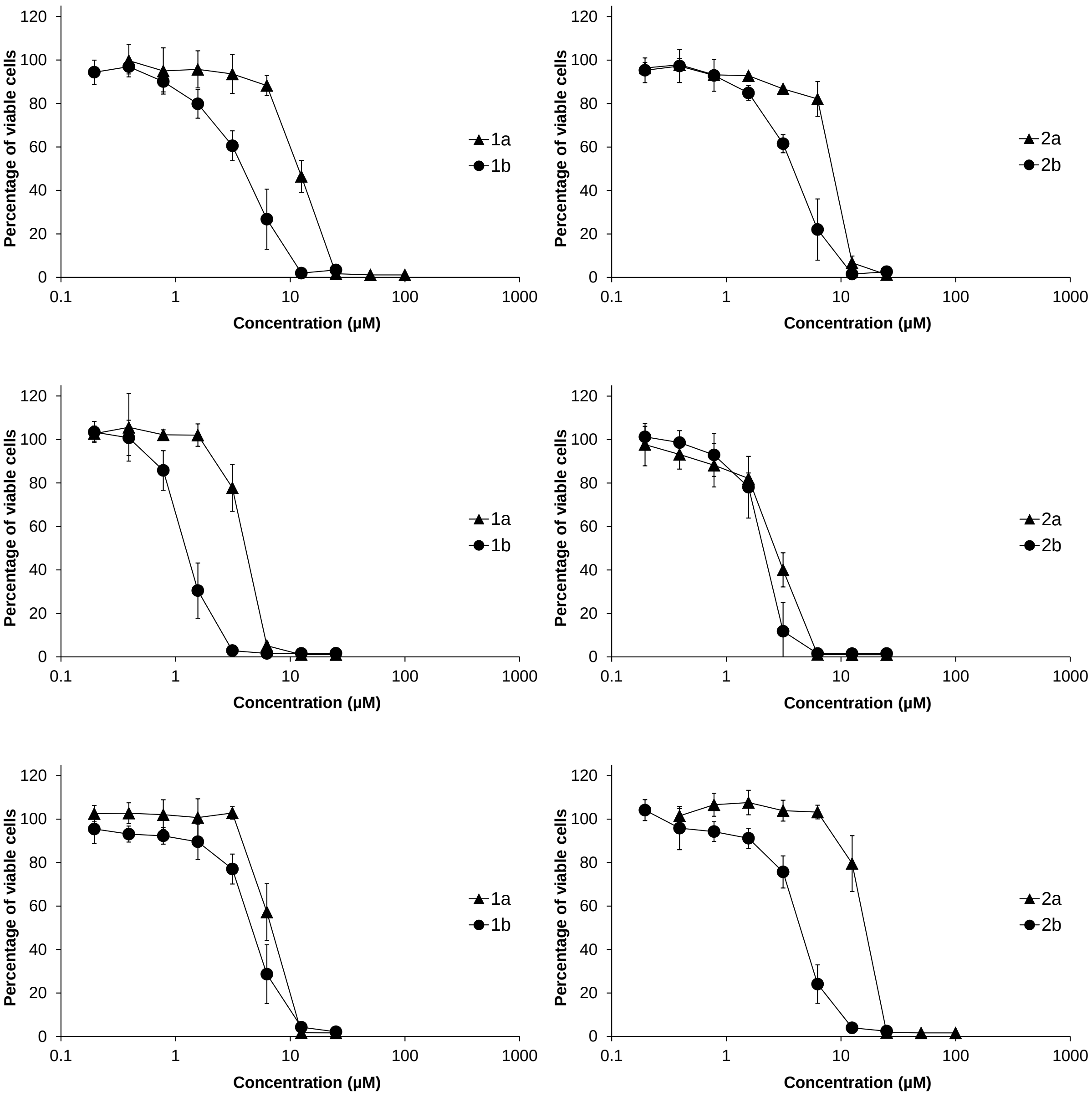

Figure S10: Concentration-effect curves of compounds 1a and 1b (left), 2a and 2b (right) in A549 (top), CH1/PA-1 (middle) and SW480 (bottom) cells, obtained by the MTT assay (exposure: 96 h). Values are means ± standard deviations from at least three independent experiments.

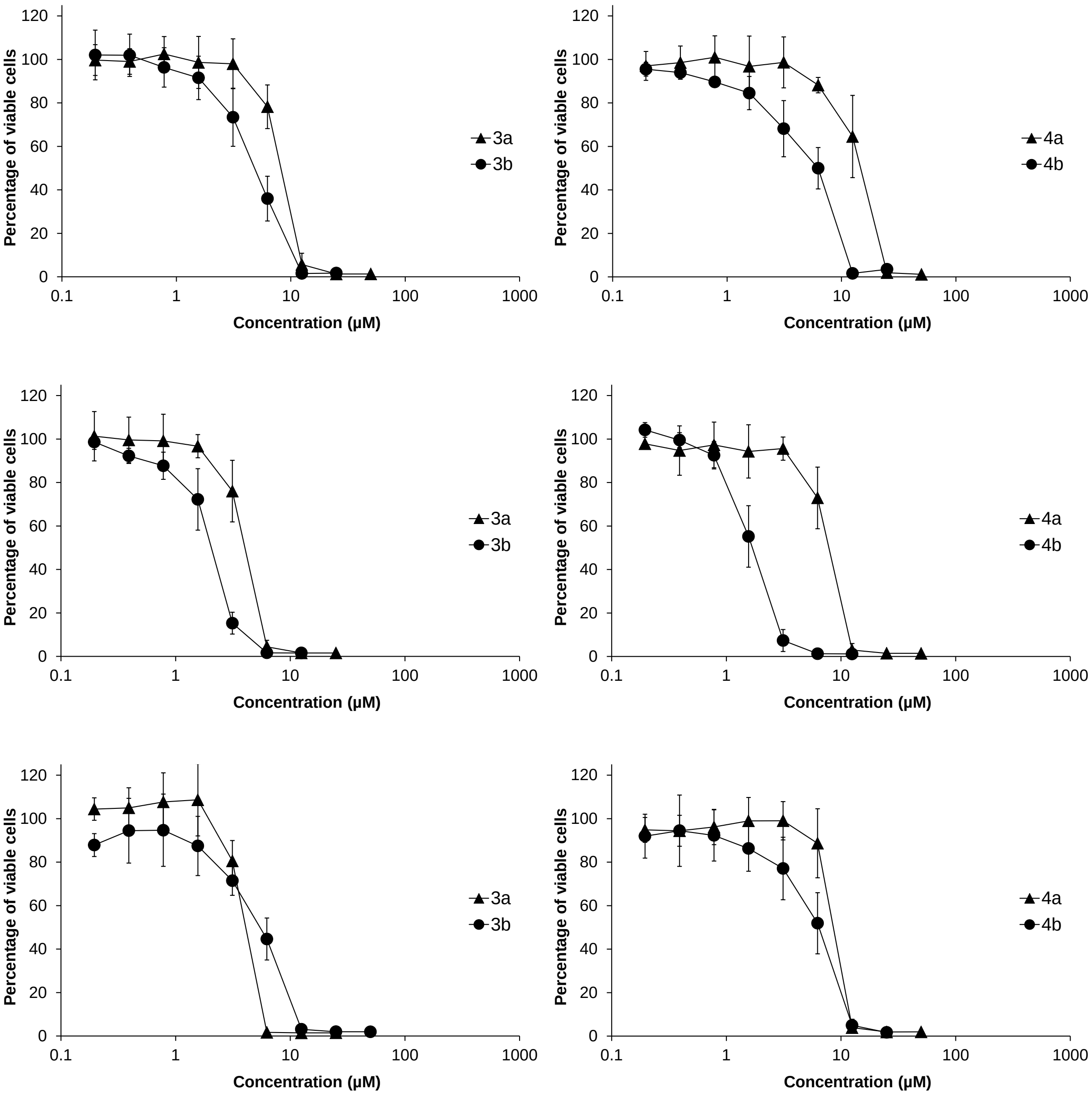

Figure S11: Concentration-effect curves of compounds 3a and 3b (left), 4a and 4b (right) in A549 (top), CH1/PA-1 (middle) and SW480 (bottom) cells, obtained by the MTT assay (exposure: 96 h). Values are means ± standard deviations from at least three independent experiments.

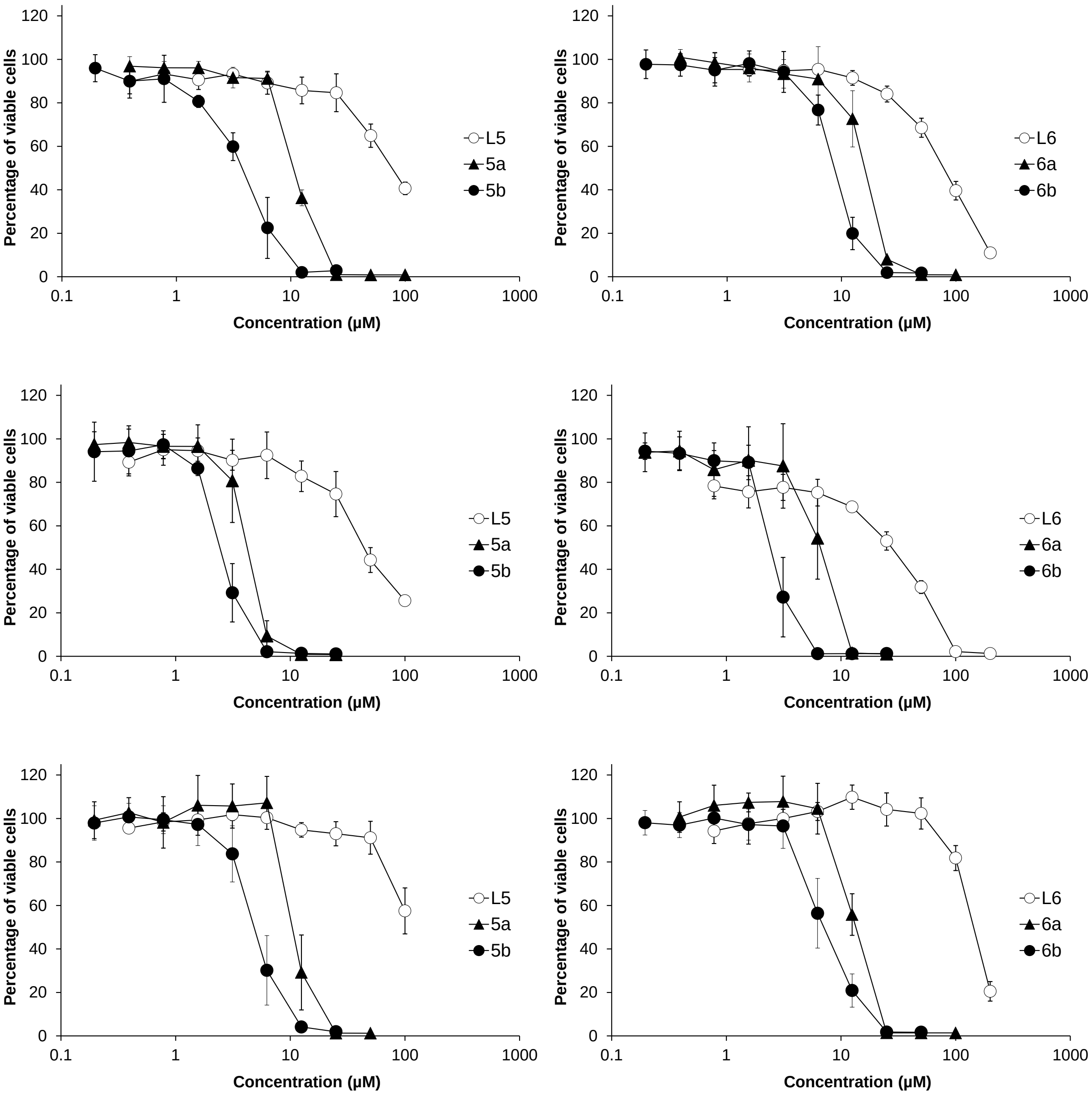

Figure S12: Concentration-effect curves of compounds L5, 5a and 5b (left), L6, 6a and 6b (right) in A549 (top), CH1/PA-1 (middle) and SW480 (bottom) cells, obtained by the MTT assay (exposure: 96 h). Values are means ± standard deviations from at least three independent experiments.

4.1 FACS Studies

Table S10. Apoptosis induction by 2a, 2b, 6a and 6b after 24 h in SW480 cells, analyzed by the flow-cytometric AV/PI assay differentiating viable (AV–/PI–), early apoptotic (AV+/PI–), late apoptotic (AV+/PI+) and necrotic (AV–/PI+) cell fractions. Percentages are means ± standard deviations from three independent experiments.

Compound	Conc. [µM]	viable cells [%]	early apoptotic cells [%]	late apoptotic cells [%]	necrotic cells [%]
neg. control	0	95.4 ± 1.0	2.1 ± 0.8	2.2 ± 0.4	0.4 ± 0.2
pos. control	50	6.4 ± 5.5	1.8 ± 2.6	87.7 ± 5.9	4.1 ± 2.9
**2a**	5	91.6 ± 2.6	2.0 ± 1.6	5.7 ± 1.4	0.8 ± 0.3
	10	75.9 ± 2.2	13.7 ± 2.5	8.9 ± 0.7	1.6 ± 0.8
	20	69.3 ± 12.3	2.3 ± 0.9	22.4 ± 8.1	6.0± 5.1
	40	1.0 ± 0.2	3.0 ± 0.2	85.1 ± 2.3	10.9 ± 1.9
**2b**	5	95.4 ± 1.0	1.8 ± 0.7	2.5 ± 0.5	0.3 ± 0.2
	10	95.9 ± 1.0	1.0 ± 0.4	2.8 ± 0.6	0.3 ± 0.2
	20	95.3 ± 1.4	0.9 ± 0.4	3.4 ± 0.9	0.4 ± 0.3
	40	94.3 ± 1.3	1.0 ± 0.5	4.3 ± 1.0	0.4 ± 0.1
**6a**	5	95.6 ± 1.6	1.8 ± 1.2	2.1 ± 0.5	0.5 ± 0.1
	10	94.6 ± 1.0	2.3 ± 1.2	2.7 ± 0.3	0.6 ± 0.3
	20	71.0 ± 3.1	17.4 ± 3.4	10.6 ± 1.0	1.0 ± 0.5
	40	80.7 ± 3.3	7.1 ± 1.4	10.9 ± 2.0	1.4 ± 0.1
**6b**	5	95.3 ± 1.2	2.1 ± 1.2	2.2 ± 0.3	0.4 ± 0.3
	10	94.6 ± 1.1	2.2 ± 1.0	2.9 ± 0.3	0.4 ± 0.2
	20	93.9 ± 0.6	1.8 ± 0.7	3.8 ± 0.2	0.5 ± 0.2
	40	85.3 ± 1.0	3.2 ± 0.1	10.7 ± 1.0	0.8 ± 0.4

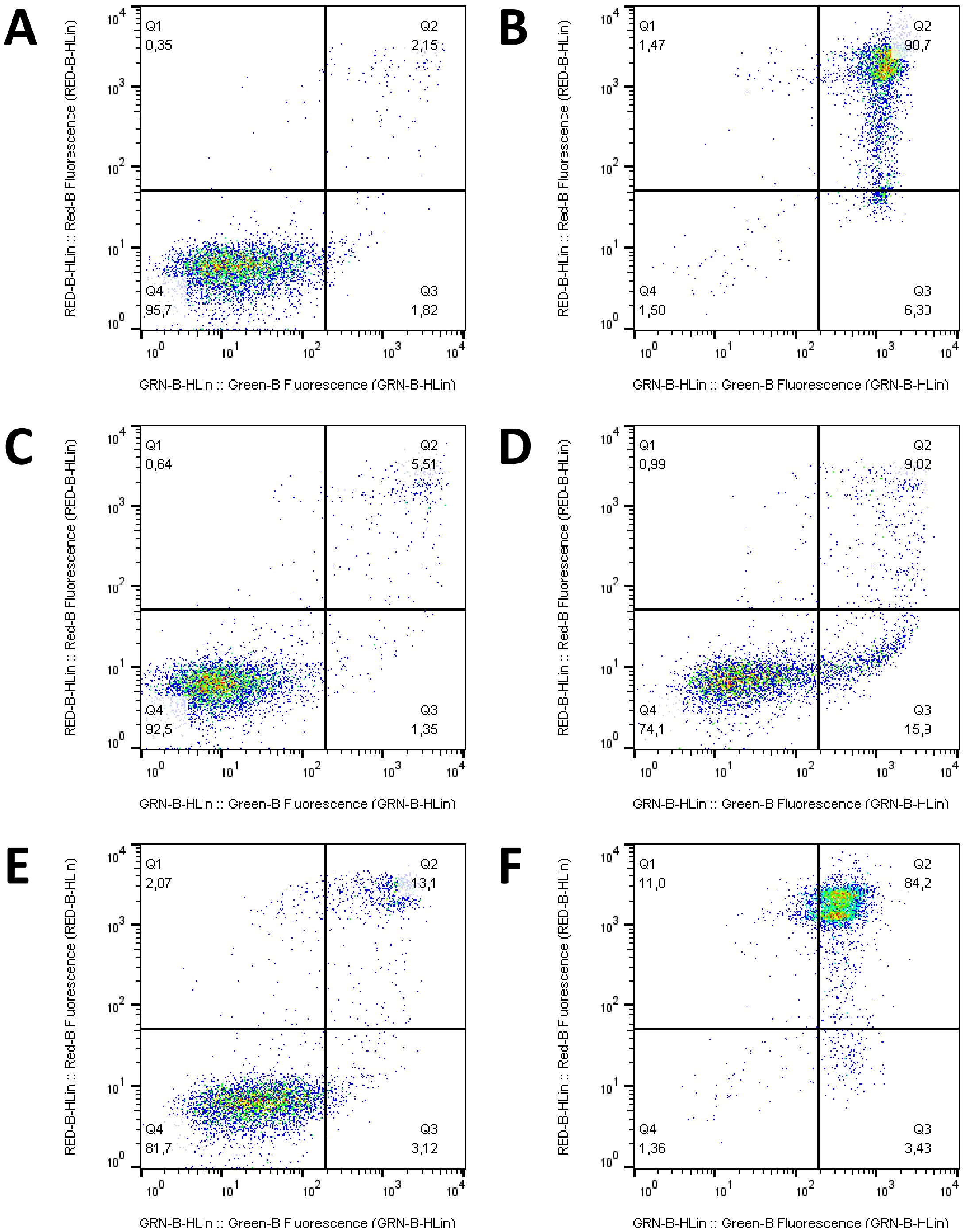

Figure S13. Apoptosis induction by 2a after 24 h in SW480 cells, analyzed by the flow-cytometric AV/PI assay. Dot plots represent necrotic (Q1: AV–/PI+), late apoptotic (Q2: AV+/PI+), early apoptotic (Q3: AV+/PI–) and viable (Q4: AV–/PI–) cell fractions (percentages indicated correspondingly) from a selected experiment: (A) untreated control; (B) positive control (50 µM of Pt complex KP1998(Scaffidi-Domianello et al., 2012)); (C) 5 µM, (D) 10 µM, (E) 20 µM and (F) 40 µM of 2a.

4.2 Cellular Uptake Studies

Table S11. Instrumental parameters for ICP-MS analysis. (Klose et al., 2017)

	**ICP-MS Agilent 7500ce**
**RF power [W]**	1560
**Cone material**	Nickel
**Carrier gas [L/min]**	0.92–0.97
**Make up gas [L/min]**	0.22–0.27
**Plasma gas [L/min]**	15
**Monitored isotopes**	^{102}Ru, ^{115}In, ^{185}Re, ^{189}Os
**Dwell time [s]**	0.3
**Number of replicates**	10

For Ru, In served as internal standard, while Re was used as internal standard for Os.

5 References

Klose, M.H.M., Hejl, M., Heffeter, P., Jakupec, M.A., Meier-Menches, S.M., Berger, W., et al. (2017). Post-digestion stabilization of osmium enables quantification by ICP-MS in cell culture and tissue. *Analyst* 142(13)**,** 2327-2332. doi: 10.1039/c7an00350a.

Scaffidi-Domianello, Y.Y., Legin, A.A., Jakupec, M.A., Roller, A., Kukushkin, V.Y., Galanski, M., et al. (2012). Novel Oximato-Bridged Platinum(II) Di- and Trimer(s): Synthetic, Structural, and in Vitro Anticancer Activity Studies. *Inorganic Chemistry* 51(13)**,** 7153-7163. doi: 10.1021/ic300148e.
